# Polarity-Induced Reactive Wetting: Spreading and Retracting Sessile Water Drops

**DOI:** 10.1021/acs.langmuir.4c01085

**Published:** 2024-06-14

**Authors:** William S. Y. Wong, Mariia S. Kiseleva, Abhinav Naga

**Affiliations:** † Department of Applied Physics, School of Science, Aalto University, FI-02150 Espoo, Finland; ‡ Department of Physics, Durham University, Durham DH1 3LE, U.K.

## Abstract

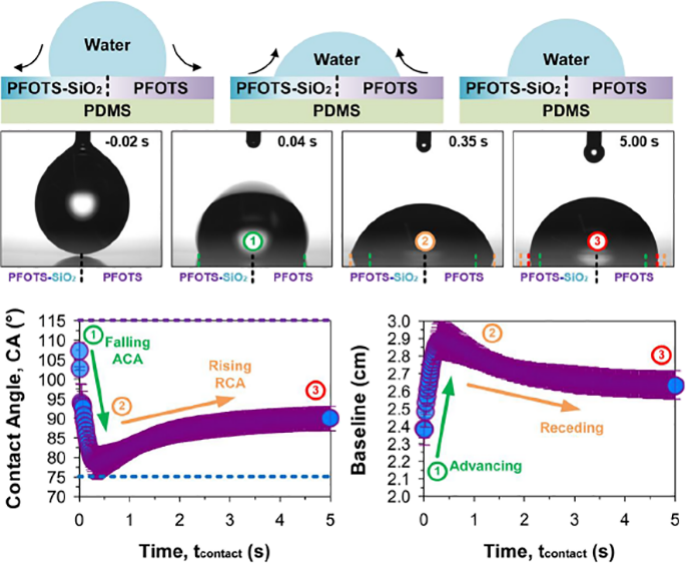

Wetting is typically defined by the relative liquid to solid surface tension/energy, which are composed of polar and nonpolar subcontributions. Current studies often assume that they remain invariant, that is, surfaces are wetting-inert. Complex wetting scenarios, such as adaptive or reactive wetting processes, may involve time-dependent variations in interfacial energies. To maximize differences in energetic states, we employ low-energy perfluoroalkyls integrated with high-energy silica-based polar moieties grown on low-energy polydimethylsiloxane. To this end, we tune the hydrophilic-like wettability on these perfluoroalkyl-silica-polydimethylsiloxane surfaces. Drop contact behaviors range from invariantly hydrophobic at ca. 110° to rapidly spreading at ca. 0° within 5 s. Unintuitively, these vapor-grown surfaces transit toward greater hydrophilicity with increasing perfluoroalkyl deposition. Notably, this occurs as sequential silica-and-perfluoroalkyl deposition also leaves behind embedded polar moieties. We highlight how surfaces having such chemical heterogeneity are inherently wetting-reactive. By creating an abrupt wetting transition composed of reactive and inert domains, we introduce spatial dependency. Drops contacting the transition spread before retracting, occurring over the time scale of a few seconds. This phenomenon contradicts current understanding, exhibiting a uniquely (1) decreasing advancing contact angle and (2) increasing receding contact angle. To explain the behavior, we model such time- and space- dependent reactive wetting using first order kinetics. In doing so, we explore how reactive and recovery mechanisms govern the characteristic time scales of spreading and retracting sessile drops.

## Introduction

The wettability of surfaces is often defined by the concept of a contact angle. This angle, θ, is conventionally defined at the three-phase contact line, at which the solid–liquid, liquid–gas, and solid–gas interfaces meet. Mathematically, the contact angle is related to the surface energies of these 3 interfaces according to Young’s law, . One key assumption underlying this understanding is the presence of a thick inert solid surface; that is, the solid has an infinite depth which defines a uniform and invariant surface energy during liquid contact.^1^ In reality, γ_SL_ can be dynamic during and after liquid contact, particularly in adaptive or reactive wetting.^2−4^ Reactive wetting has so far been explored primarily with liquid metals drops on metal substrates,^4,5^ often demonstrated at high temperatures.^4^ Numerous models are proposed for describing the phenomenon,^4^ with focus centered on hydrodynamics,^6−8^ molecular kinetics,^8^ combined models,^9^ and roughness-induced imbibition.^10^ Within these experimental-to-theoretical works,^4,6−9^ one predominant observation persists: Sessile drops spread unidirectionally towards a final equilibrium state but does not retract without external energy input such as heat,^4,11^ applied voltage,^12^ or the use of so-termed autophobic liquids.^13−16^

In this work, we attempt to achieve and understand reactive wetting by layering drastically different surface chemistries, hence tuning the effective interfacial energies. This design aims to provide a description towards surface-induced wetting reactivity with common liquids: water and partially polar liquids. First, sequential chemical vapor deposition of hydrophobic perfluoroalkyls and hydrophilic silica is used to create wetting-reactive surfaces. These are assessed alongside wetting-inert controls comprising only perfluoroalkyls. The layers are combined on a hydrophobic and largely nonpolar polydimethylsiloxane (PDMS) substrate base. The unexpected wetting-reactive behavior in the former occurs due to two sequential but nonexclusive possibilities: (1) perfluoroalkyls are intermixed with water-soluble polar moieties that remain after chemical vapor deposition. Upon time-delayed wetting-breakthrough of the perfluoroalkylated layer, (2) silica sublayers with polar or even electrostatic properties will then rapidly absorb the contacting liquid. To confirm polarity-induced contributions, we probed the wetting behaviors of these surfaces using water, nonpolar oil (i.e., hexadecane), and a polar solvent (i.e., tetrahydrofuran). Experimental findings with both flat and microstructured surfaces show that polar-to-polar interactions likely drive the initial time-dependent spreading dynamics.

Second, to achieve complex reactive wetting dynamics showcasing bidirectional wetting (i.e., spreading and retracting), we designed a surface with an abrupt wetting transition that imparts spatial dependency. The wetting transition is composed of split-domains bearing wetting-reactive and inert surfaces (at millimetric-scale). Domains are separated by a sharp gradient. As a sessile drop contacts the wetting transition, it initially spreads rapidly outwards with a falling advancing contact angle. Driven by the wetting-reactive domain, the spreading drop overextends into the wetting-inert domain. At a critical threshold, this results in a reversal of driving force. This reversal leads to contact line retraction and a rising receding contact angle. To understand spreading-retracting dynamics, we model the time- and spatial-dependent phenomenon using first order kinetics. We estimate the characteristic time scales of spreading and retracting and correlate them to mechanistic surface reactivity and recovery.

Our findings demonstrate the versatility of surface-directed reactive wetting, where wetting and dewetting modes may be triggered on demand. In the state-of-the-art, spatially and temporally dependent dynamic wetting often involves external energy input, using magnetism,^17^ thermal,^11,18^ or potential difference.^19^ Contrasting this, our work exploits wetting-driven surface energy variations to drive wetting behaviors. Wettability changes by virtue of wetting contact are useful for applications exploiting wetting history, such as liquid-gating membranes^20−23^ or improving bioadhesion of hydrophobic materials with biological fluids or tissues.^24−26^

## Experimental Section

### Synthesis of Surfaces

#### Synthesis of Macroscopically Flat and Model Microstructured Surfaces

Model micropillar arrays (60 μm width, 130 μm wall-to-wall distance, at 90% gas fraction) were designed in KLayout as negative molds (photoresists forming pits, as.gds files) before fabrication via maskless lithography (MLA) methods in a cleanroom (Micronova, Aalto University). 4-in. silicon wafers (J14125, Siegert Wafer, ⟨100⟩) were used as the substrate, with 10 mL of SU8-50 (Microchem) as the photoresist. Silicon wafers were first heated in a clean, dry oven at 120 °C for 2 h for dehydration, before spin-coating SU-8-50 at 500 rpm for 5 s (Ramp: 200 rpm/s) and 1500 rpm for 30 s (Ramp: 300 rpm/s). Wafers were then prebaked (Programmable hot plate, RHS) at 65 °C for 15 min (Ramp: 21.6 °C/min) and 95 °C for 15 min (Ramp: 10 °C/min). Baked wafers are then cooled and loaded onto the MLA 150 (Heidelberg Instruments) and exposed using an optimal setting of −17 defocus with 300 mJ/cm^2^. Exposed wafers are then postbaked at 95 °C for 12 min (Ramp: 19 °C/min) and cooled at 3.75 °C/min to room temperature. Wafers are then cooled down and immersed in a bath of developer solution for 20 min with swirling at 5 min intervals. Wafers are retrieved and washed using isopropanol and dried with a nitrogen air gun. A reactive ion etching (RIE) program (Oxford Instruments, Plasma RF generator) is used to deposit a thin layer (30 nm) of fluoropolymer using CF_6_ at 99.5 cm^3^/min over 10 min with a DC bias of 80 V (Chamber pressure at 250 mTorr). Negative resist-coated wafers are retrieved from the cleanroom and templated using polydimethylsiloxane (PDMS, Sylgard 184). PDMS was prepared using cross-linked Sylgard 184 PDMS, mixed at a 1:10 weight ratio (1:10 g) of cross-linker-to-vinyldimethylsiloxane, respectively, in a 100 mL cup, and stirred vigorously before evacuation in a clean desiccator to remove bubbles. Approximately 40 mL is poured onto a 4-in. wafer in Petri dish (20 cm diameter) before curing in an oven at 80 °C for 3 h. The soft templated PDMS is then cut with a scalpel and peeled off of the negative mold. Macroscopically flat PDMS is fabricated under the same conditions, without the use of lithographically synthesized microstructured templates.

#### Chemical Vapor Deposition of Polar Silica Layers

To create polar silica layers, a technique for creating silica shells is used. This reaction takes place via tetraethylorthosilicate (TEOS, 99.9%, Alfa Aesar) and 30% ammonium hydroxide (NH_4_OH, 30%, Sigma-Aldrich). Pristine PDMS surfaces (macroscopically flat or microstructured) were placed into the center of a desiccator (20 cm diameter, *V* = 4.2 L), where 2 mL of TEOS and 2 mL of NH_4_OH were deposited on the perimeter edge (ca. 8 cm from the samples and 16 cm from each other). The desiccator was then evacuated to 50 mbar and kept for 3 h. The ambient lab environment was at ca. 10–20% relative humidity, 20 °C. This culminates in the PDMS-SiO_2_ variant. This method is known for creating very conformal and smooth surfaces, which was confirmed by scanning electron microscopy.

#### Chemical Vapor Deposition of Perfluoroalkyls

In addition to both pristine PDMS and PDMS-SiO_2_ surface variants, controls were included to improve our understanding of the reaction. Silicon wafers (prime grade single-side polished silicon, thickness of 525 ± 10 μm and (100) orientation) were also used as substrates. Silicon substrates were cut to ca. 15 mm × 15 mm and then cleaned with ethanol. To activate these surfaces prior to functionalization, substrates were oxygen plasma treated for 10 min, at 100% power (Diener Electronic, PCCE, 300W). To hydrophobicize the target surfaces (control Si wafers, flat, and flat/microstructured PDMS and PDMS-SiO_2_), 1*H*,1*H*,2*H*,2*H*-perfluorooctyltrichlorosilane (PFOTS, 97%, Sigma-Aldrich) is used to create the perfluoroalkylated layer. Activated surfaces were placed into a desiccator (20 cm diameter, *V* = 4.2 L) at ca. 8 cm from the center, where 150 μL of PFOTS was deposited. The desiccator was then evacuated to 50 mbar for variable residence time of 1, 5, 10, 20, 30, and 60 min. 10 min of exposure is used as the model residence time for reactive wetting without inducing superspreading. For the silicon wafer control, an additional condition was tested, with 500 μL of PFOTS for 30 min, the so-termed extended functionalization. The ambient lab environment was at ca. 10–20% relative humidity, 20 °C. After the reaction, all functionalized surfaces were then evacuated at 50 mbar (*in situ* without silane present) for 30 min to remove residual silanes. Functionalized surfaces were left to equilibrate with the ambient air environment (*T* = 20 °C, humidity = 10–20%) for at least 1 day before testing. The thicknesses of the perfluoroalkylated layers were assessed using ellipsometry.

### Characterization of Surfaces

#### Wetting Analysis

Wetting was assessed using the measurement of sessile-drop-based static contact angles (CAs), by placing and averaging 3–6 drops of water (5 μL) and tetrahydrofuran/hexadecane (3 μL) on three cross-batch sample surfaces. This was delicately performed to ensure repeatability, with drops extruded from needle tips (30 G) before the drops were moved down to the surface at 0.1 mm/s until contact. Upon touch contact, the needle is then retracted, allowing the drop to detach onto the surface (if not already so). The behavior of the drop is then recorded at 69 frames per second for up to 60 s to capture any dynamics. Dynamic images were recorded using a Biolin Attension Theta Goniometer (Finland) with a Navitar camera (1-60135, Canada). Camera settings were exposure (3600), gamma (2000), and gain (0), at a magnification of 0.7×. The CA and SA were computed by a commercially available (OneAttension) program. Data are presented as mean ± standard deviations (SD) or standard errors (SE). While the authors acknowledge the importance of dynamic methods such as roll-off/sliding angle (SA) or contact angle hysteresis (CAH) tests, reactive wetting surfaces often experience a dynamic wetting behavior where the contact angle changes rapidly within the first seconds of contact. Therefore, a delicately controlled sessile drop method (as described above) is the only method appropriate for wetting dynamics that occur within a few seconds. Due to the time delays present in SA and CAH measurements, they are not suitable for assessing reactive wetting surfaces. Nonetheless, CAH measurements were performed (see Supporting Information) on PFOTS on silicon wafers under both limited (10 min) and extended (30 min) functionalization to provide broader appreciation of the perfluoroalkylation process. 10 μL of water was deposited onto the surfaces at 1 μL/s. Thereafter, to expand the drop and move the contact line outwards, another 20 μL was deposited at 0.05 μL/s. The advancing contact angle is taken at the point where the contact line begins a smooth motion. At a drop size of 30 μL, the drop is then contracted by withdrawing 10 μL at 0.05 μL/s, to move the contact line inwards. Images are recorded at 1.4 fps.

#### Surface Analysis

Samples were analyzed via a Zeiss Sigma VP scanning electron microscope (SEM) at an accelerating voltage of 2–3 kV with a working distance of 2–3 mm. Aperture: 30 μm, detector: InLens. All surfaces were coated with Ir (10 nm), Au–Pd (10 nm), and W (10 nm) by sputter-coating (Leica EM ACE 600) before analysis. No significant differences were noted. To provide a qualitative analysis of F distribution on the surfaces, top-down profiles were also analyzed using the SEM-EDX mode (Oxford Instruments), at an accelerating voltage of 8 kV with a working distance of 8.5 mm at 50× magnification, resolution of 256 over 256 frames (Capture time: 30 min). The EDX mapping is used to qualitatively illustrate the presence and absence of perfluoroalkylated groups (F).

#### Ellipsometry Analysis

Coating thicknesses were determined using a spectroscopic ellipsometer (M2000UI, JA Woollam, USA). Measurements were performed in the spectral range from 246 to 1689 nm at a 75° angle from the surface normal with an acquisition time of 5 s in “High accuracy mode”. The data was obtained and analyzed using the device software package (CompleteEASE ver. 6.53, JA Woollam, USA). Measurements for each surface variant were made in at least in three different spots.

#### X-ray Photoelectron Spectroscopy

The X-ray photoelectron spectroscopy (XPS) measurements were made using a Kratos Axis Ultra system (Kratos Analytical, UK), equipped with a monochromatic Al Kα X-ray source. High resolution and survey spectra were obtained at step sizes of 0.01 and 0.5 eV, respectively. All measurements were performed within a 5 mm × 5 mm analysis area. The C 1s peaks were used for calibration, giving an uncertainty of up to 0.1 eV. The binding energy range (BE) from 0 to 1500 eV was selected. The XPS spectra were processed using CasaXPS software. The XPS background was obtained by the Shirley method.

## Results and Discussion

### Wetting Reactivity of Chemical Vapor-Deposited Perfluoroalkylated Silica

**Figure 1:**
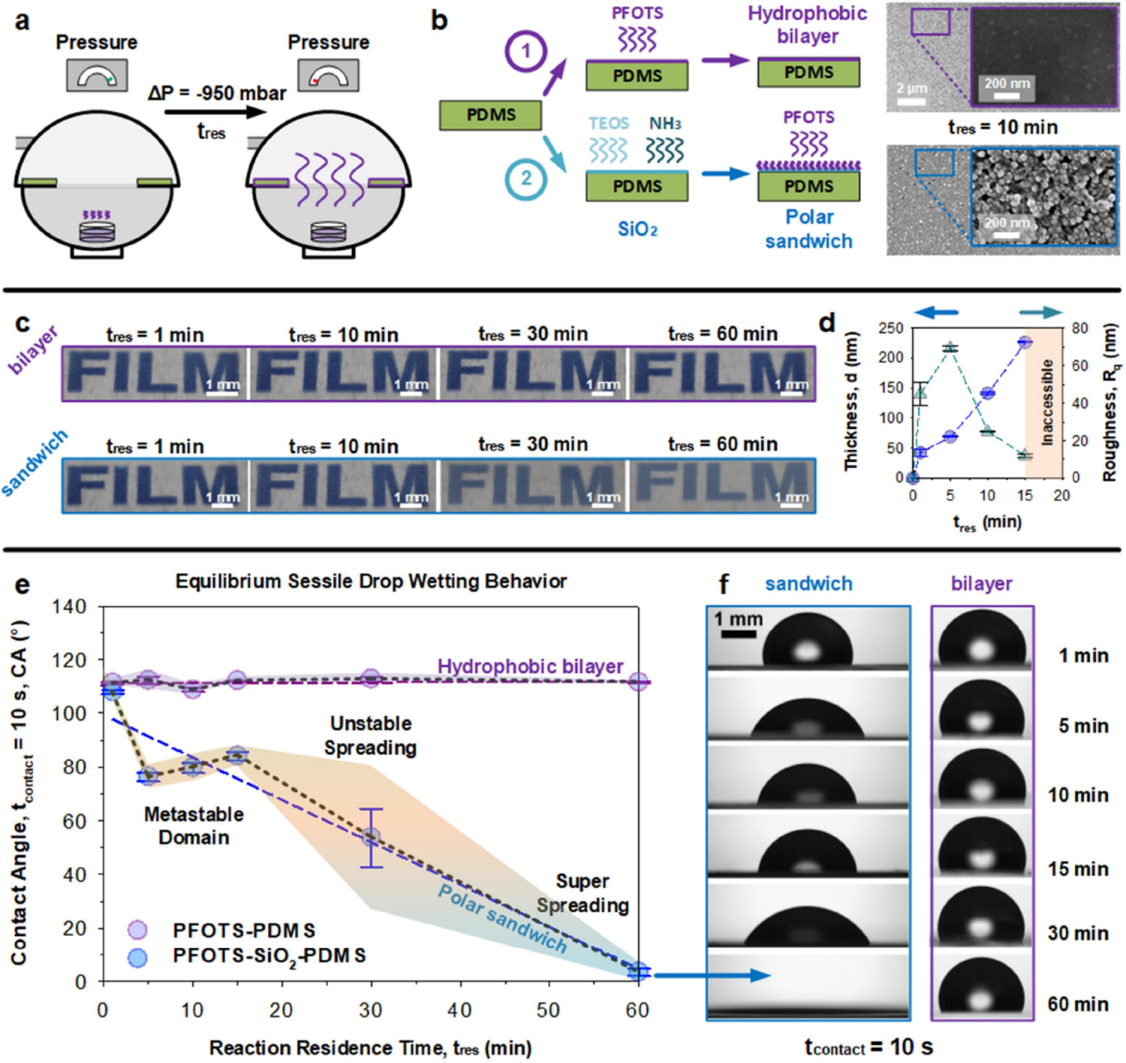
Unexpected reactive wetting of perfluoroalkylated silica layers. (a) Chemical vapor deposition of volatile precursors (PFOTS and TEOS-NH_3_) is used to create (b) hydrophobic bilayer (control: PFOTS–PDMS) and the polar sandwich (PFOTS–SiO_2_–PDMS). The former is smoother, while the latter possesses nanoroughness. (c) The optical opacity of the polar sandwich presented alongside analytical measurements of (d) ellipsometric thickness, d and r.m.s. roughness, *R*_q_. (e) Sessile drop wetting of inert hydrophobic bilayer (purple) and wetting-reactive polar sandwich (blue) with respect to reaction residence time (of PFOTS), *n* = 6 ± SE (bars), and ± SD (shaded domains). (f) Hydrophobic bilayer is wetting-inert, while the polar sandwich becomes increasingly wetting-reactive beyond short deposition times (>1 min).

In the development of liquid-repellent or super liquid-repellent surfaces, chemical vapor deposition (CVD) is one of the primary means for surface functionalization. This includes placing a volatile precursor in a reaction chamber followed by either thermal heating^27^ and/or evacuation.^28−31^ This creates a chemical vapor that functionalizes surface-active substrates within the reaction chamber (Figure 1a). In this work, two surface types are configured on a hydrophobic PDMS substrate. First, as a control, perfluoroalkyls are deposited as a layer onto PDMS via 1*H*,1*H*,2*H*,2*H*-perfluorooctyltrichlorosilane (PFOTS) at 50 mbar (*P*_PFOTS_ = 0.7 ± 0.5 mbar) at 20 °C. This is termed the hydrophobic bilayer which is nonpolar and wetting-inert (PFOTS–PDMS, Figure 1b). Second, for wetting-reactive surfaces, silica is first deposited onto PDMS using tetraethylorthosilicate (TEOS) and aqueous ammonia (30% NH_3_ (aq)) as a catalyst, at 50 mbar for 3 h. Thereafter, perfluoroalkyls (PFOTS) are deposited as a second layer onto the silica at 50 mbar (*P*_PFOTS_ = 0.7 ± 0.5 mbar) at 20 °C. This surface is designed to be partially polar and is termed the polar sandwich (PFOTS–SiO_2_–PDMS, Figure 1b). In both instances, oxygen plasma activation was performed before PFOTS deposition (300 W, 10 min). The former is very smooth, while the latter is notably rougher at the nanometer scale (Figure 1b, insets). The increased roughness of the latter is expected due to the higher reactivity of silica towards trichlorosilanes (vs PDMS) under identical reaction conditions. Notably, increased roughness is not induced during the vapor deposition of the silica layer. Scanning electron microscopy at high magnification shows a very smooth surface, akin to pristine PDMS (see Figure S1). In the slightly rougher perfluoroalkylated layer of the polar sandwich, the presence of roughness on a wetting-inert hydrophobic surface would generally lead to higher contact angles. However, we will observe, in the following sections, that the reverse occurs: It becomes more wettable, driven primarily by polarity-induced reactive wetting.

The residence time (*t*_res_) of the PFOTS CVD is tuned from 1 to 60 min to increase the extent of deposited perfluoroalkylated moieties (Figure 1c). Synthesis parameters for the silica layer is kept constant. Notably, the polar sandwich became increasingly opaque with higher *t*_res_, contrasting with that of the bilayer (staying transparent). In the following sections, a *t*_res_ of 10 min is chosen to create polar sandwich surfaces. In this configuration, we do not observe significant capillary-like wicking behaviors, unlike those at higher *t*_res_. Therefore, this limits the influence of geometrical contributions (roughness, Figure 1d) that may also influence dynamic wetting behaviors. Further experimental details can be found in the Experimental Section (and Figure S2).

Wetting of any surface depends on the nature of liquid to surface interaction.^1,32−35^ Water is polar (δ_p_ = 16, δ_d_ = 15.6, δ_H_ = 42.3) and readily forms a finite measurable^36^ contact angle with a range of surface chemistries. Hence, it serves as an ideal probe liquid for understanding polarity-induced wetting between our different surface chemistries (hydrophobic bilayer vs polar sandwich). For the hydrophobic bilayer, polar-to-polar interactions are negligible. Therefore, wetting depends primarily on the nonpolar properties of the liquid and the surface. For the polar sandwich, polar-to-polar interactions can dominate the wetting behaviors. In the following experiments, a very slow sessile drop test (0.1 mm/s contact velocity) was performed to help capture wetting dynamics during initial wetting (*t*_contact_ < 1 s) and at quasi-equilibrium (*t*_contact_ = 10 s). On the hydrophobic bilayer, water contact angles are invariant (besides minute contact vibrations) regardless of PFOTS CVD residence time (Figure 1e, purple data, and Figure S3). In contrast to this, water contact angles on the polar sandwich varied (Figure 1e, blue data, and Figure S3) from ca. 110° down to ca. 0° at quasi-equilibrium. An unstable transition was noted at *ca. t*_contact_ = 30 min. Collectively, these sessile drop experiments show how wetting-reactive behaviors (“hydrophilic” spreading) can be achieved by embedding polar moieties within perfluoroalkylated surfaces to foster strong polar interactions. In the following section, we describe the origins of these polar moieties.

#### Mechanism: Origins of Polarity-Induced Reactive Wetting in Perfluoroalkylated Silica

**Figure 2:**
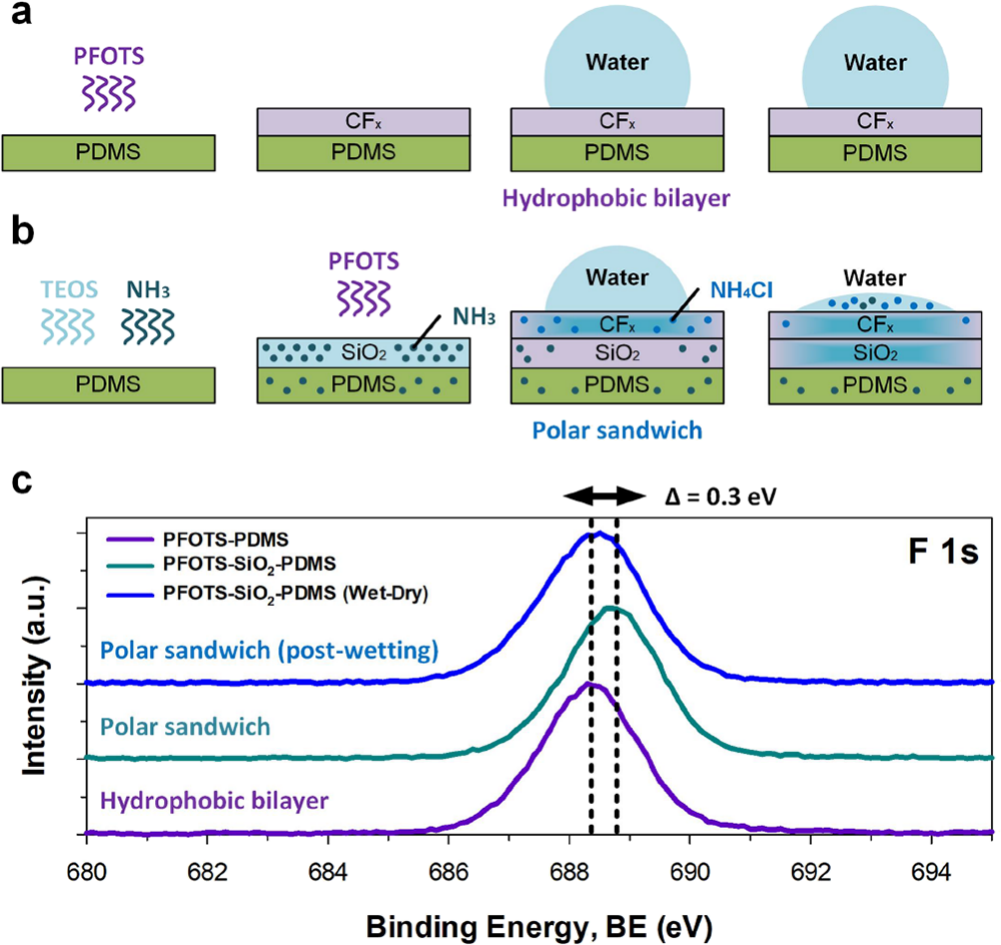
Mechanism: polarity-induced reactive wetting. (a) A hydrophobic bilayer (PFOTS–PDMS) is wetting-inert and hydrophobic. (b) A polar sandwich (PFOTS–SiO_2_–PDMS) is wetting-reactive and appears to be very hydrophilic, where contact angles are unexpectedly low despite a high fluoroalkylated content (Table 1). Upon wetting, the CVD polar side-products (NH_4_Cl) initiate drop spreading, which further spreads into the subsurface silica. (c) +0.3 eV shift in XPS (X-ray photoelectron spectroscopy) analysis suggests local changes in the chemical environment surrounding fluorine atoms, such as alterations to elemental composition.

The unexpected wettability of chemical-vapor-deposited perfluoroalkylated silica is attributed to secondary reactions triggered from byproducts following the sequential Stöber (SiO_2_) and sol–gel (PFOTS) reactions. We illustrate here how PFOTS-on-PDMS is wetting-inert and hydrophobic (Figures 1e,f and 2a). In contrast to this, PFOTS-on-SiO_2_-on-PDMS is wetting-reactive (Figures 1e,f and 2b), where the contacting water drop actively spreads. This process is driven by two mechanisms. (1) Initial wetting of the perfluoroalkylated layer is induced by the presence of soluble polar moieties (NH_4_Cl) that remain after the two-step (Stöber)-to-(sol–gel) reactions (see R1–4 below). (2) Subsequent spreading is likely further driven by the presence of sublayered silica or the mixed-layer that grows. This layer likely possesses strong polar interactions^37^ with water. Organosilane-functionalized silica is also known to possess negative zeta potentials,^38^ which can further increase electrostatic interactions. Together, this behavior defines the “reactive wetting” nature of the surface.

For the polar sandwich, the sequential chemical vapor deposition of perfluoroalkylated silica gives rise to the following secondary reactions (see R1–4 below), with the Stöber reaction byproducts (R1-2: NH_3_) reacting with sol–gel reaction byproducts (R3-4: HCl), giving rise to highly polar and water-soluble NH_4_Cl (Figure 2c) that is deposited within the film as it grows. In R4, we describe the condensation reaction as a single covalent bond to the substrate surface although multiple bonds arising from the tri-functionality is also possible.


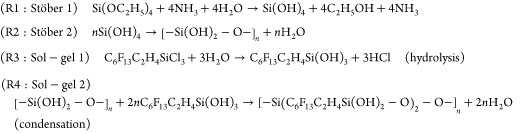


Elemental analysis of the polar sandwich surface confirms a slight shift in the peak of F 1s to higher binding energies (ca. 0.3 eV). This suggests local alterations within its immediate chemical environment such as additional atoms or chemical bonding. In this case, it is unlikely that chemical bonding is altered since the fluorine groups (−CF_2_ and −CF_3_) are largely inert. However, the chemical (and electrostatic) environment around these groups may be changed, as we further describe below. Supplementary spectra are provided in Figure S4. We attribute this change to the vapor deposition and persistent presence of highly polar and water-soluble NH_4_Cl. XPS survey spectra provide an estimate to the atomic composition of the polar sandwich surface pre- and postwetting, as highlighted in Table 1 below. This was supported by SEM-EDX (Energy-dispersive X-ray) spectroscopic mapping (Figure S5). XPS analysis confirms the presence of nitrogen and chlorine (at ca. 1:1 ratio), which is promptly removed (Table 1) during reactive wetting (which it triggers). XPS probing depth is at ca. 5 nm. However, the effective F-content (atomic %) remains largely unaffected (Table 1, XPS and Figure S5, EDX).

**Table 1:** XPS Analysis of Key Atomic Concentrations (F, Si, O, C, N, and Cl) of Polar Sandwich^a^

elements (at%)	fluorine	silicon	oxygen	carbon	nitrogen	chlorine	F/Si
polar sandwich (prewetting)	39 ± 5	7 ± 4	13 ± 1	27 ± 1	6 ± 0.5	8 ± 2	5.4
polar sandwich (postwetting)	37 ± 6	16 ± 4	22 ± 6	26 ± 5	0 ± 0	0 ± 0	2.4

a Nitrogen (N) and chlorine (Cl) are attributed to NH_4_Cl, formed via the byproducts of sequential Stöber to sol–gel reactions (NH_3_ + HCl → NH_4_Cl).

At the limit of *t*_res_ = 60 min, while the rapid spreading of water on the polar sandwich (PFOTS–SiO_2_–PDMS) is highly evident (Figure 1e, blue data, and Figure S3), replacing the bottom PDMS substrate with soda-lime glass will affect the spreading behavior. For PFOTS-SiO_2_-Glass, this effect is significantly diminished despite the presence of the iconic decay behavior in initial contact angles (see Figure S6). This suggests that PDMS itself influences the growth of subsequent layers, which can impact spreading and imbibition.

#### Initial Dynamics of Reactive Wetting by Water on Flat and Microstructured Surfaces

**Figure 3:**
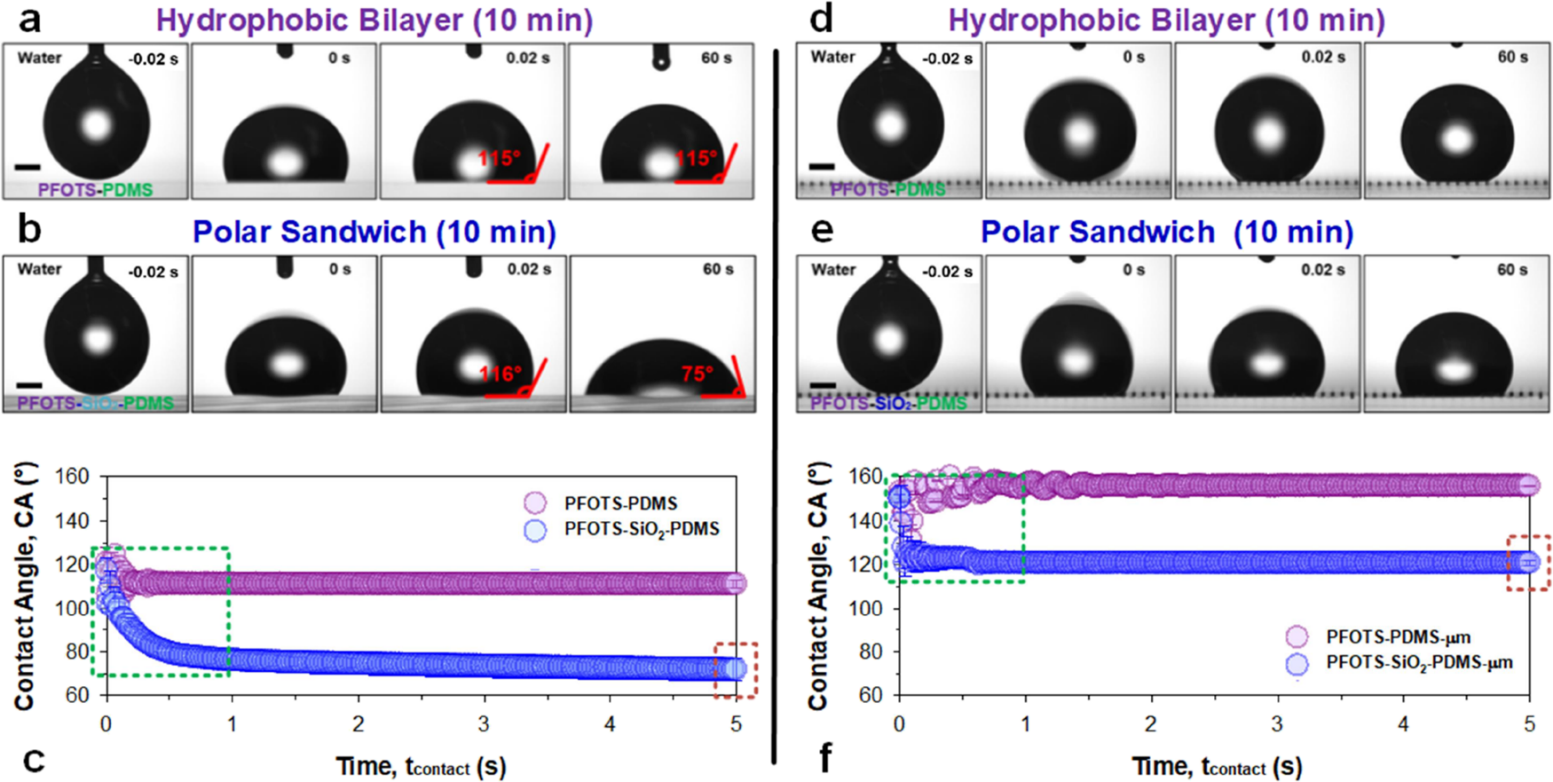
Reactive wetting by water: polar wetting probe. Wetting on (a–c) macroscopically flat and (d–f) microstructured surfaces with both (a, d) nonpolar hydrophobic bilayer and (b, e) polar sandwich configurations. Water itself is a highly polar liquid (Hansen solubility parameters, Table S1: δ_p_ = 16, δ_d_ = 15.6, δ_H_ = 42.3),^39^ which can probe the presence of polar moieties. Sessile drop wetting behavior of the hydrophobic bilayer: PFOTS–PDMS (purple) and polar sandwich: PFOTS–SiO_2_–PDMS (blue). Wetting (*n* = 3, ± SD) was measured over 60 s but plotted for the first (c, f) 5 s, where most of the dynamics occur. Scale bar: 0.5 mm.

To illustrate and further understand the effect of reactive wetting, we now compare the initial wetting dynamics of the nonpolar hydrophobic bilayer and the polar sandwich on macroscopically flat and microstructured surfaces. An intermediate *t*_res_, of 10 min, was chosen as the metastable domain for assessing reactive wetting, hence avoiding very low equilibrium contact angles (e.g., 0–10°). These surfaces were developed on macroscopically flat and microstructured surfaces (Figure 3). The perfluoroalkylated layer thickness was measured at ca. 4.3 nm (nonpolar hydrophobic bilayer, ellipsometry, Figure S7) and ca. 141 nm (polar sandwich, ellipsometry, Figure S7) respectively. The latter is expectedly rougher (*R*_q_ = 25 ± 1 nm) but without significant variations in F-content (SEM-EDX, Figure S8).

As we have observed with water, equilibrium wetting occurs significantly different on both surfaces. However, careful observation into the initial dynamics of drop contact provides further information. On macroscopically flat nonpolar hydrophobic bilayer surfaces (Figure 3a,c, purple data, PFOTS–PDMS), we see a rapid alignment between the initial and final contact angle, measured at *t*_contact_ = 0.02 or 60 s at 115°, respectively, well within the hysteresis range^28,40^ of typical PFOTS-functionalized surfaces (Figure S9). Fluctuations in measured contact angles (Figure 3c, purple data, PFOTS–PDMS) exist due to the drop vibration after detachment.

On macroscopically flat polar sandwich surfaces (Figure 3b,c, blue data, PFOTS–SiO_2_–PDMS), an exponential decrease in contact angle occurs within the first 1 s of *t*_contact_. For instance, the initial contact angle at *t*_contact_ = 0.02 s matches that of the hydrophobic bilayer at ca. 116°, but a rapid decrease to ca. 76° (Δ = −40°) takes place over the next 1 s (Figure 3b,c, blue data, PFOTS–SiO_2_–PDMS). This initial matching of contact angles between both variants indicates that water encounters similar surface energies (perfluoroalkyl) in the beginning before diverging within the first second of contact. The reactive wettability of the polar sandwich induces this bifurcated wetting behavior (see Video M1).

Following this, we performed the sessile drop experiment on model microstructured surfaces (pillar width of 60 μm, spacing of 130 μm, height of 80 μm). Again, the initial contact angle on both the nonpolar hydrophobic bilayer and the polar sandwich remains high (ca. 150°). However, upon release of the drop (needle retraction at 0.1 mm/s), it remains stable with the nonpolar hydrophobic bilayer (Figure 3d,f, purple data, PFOTS–PDMS) while collapsing into the microstructures with the polar sandwich (Figure 3e,f, bottom panel, blue data, PFOTS–SiO_2_–PDMS). The transition with the latter occurs rapidly over the course of just *t*_contact_ = 0.04 s (Δ = −30°) and reaches an equilibrium with a pinned contact line (see Video M2).

#### Reactive Wetting by Nonpolar or Polar Liquids on Flat and Microstructured Surfaces

To supplement our understanding behind wetting of the hydrophobic bilayer and the polar sandwich configurations, we attempt the same sessile drop experiments using nonpolar and polar liquids with low surface tensions. The combination of both macroscopically flat and microstructured surfaces is analyzed. Hexadecane (δ_p_ = 0, δ_d_ = 16.3, δ_H_ = 0) and tetrahydrofuran (δ_p_ = 5.7, δ_d_ = 16.8, δ_H_ = 8) represent the respective nonpolar and polar liquid variants (see Table S1).^39^ The four combinations of nonpolar and polar liquids vs nonpolar (hydrophobic bilayer) and polar (polar sandwich) surfaces will therefore be fully assessed.

##### Nonpolar Liquid (Flat Surfaces)

**Figure 4:**
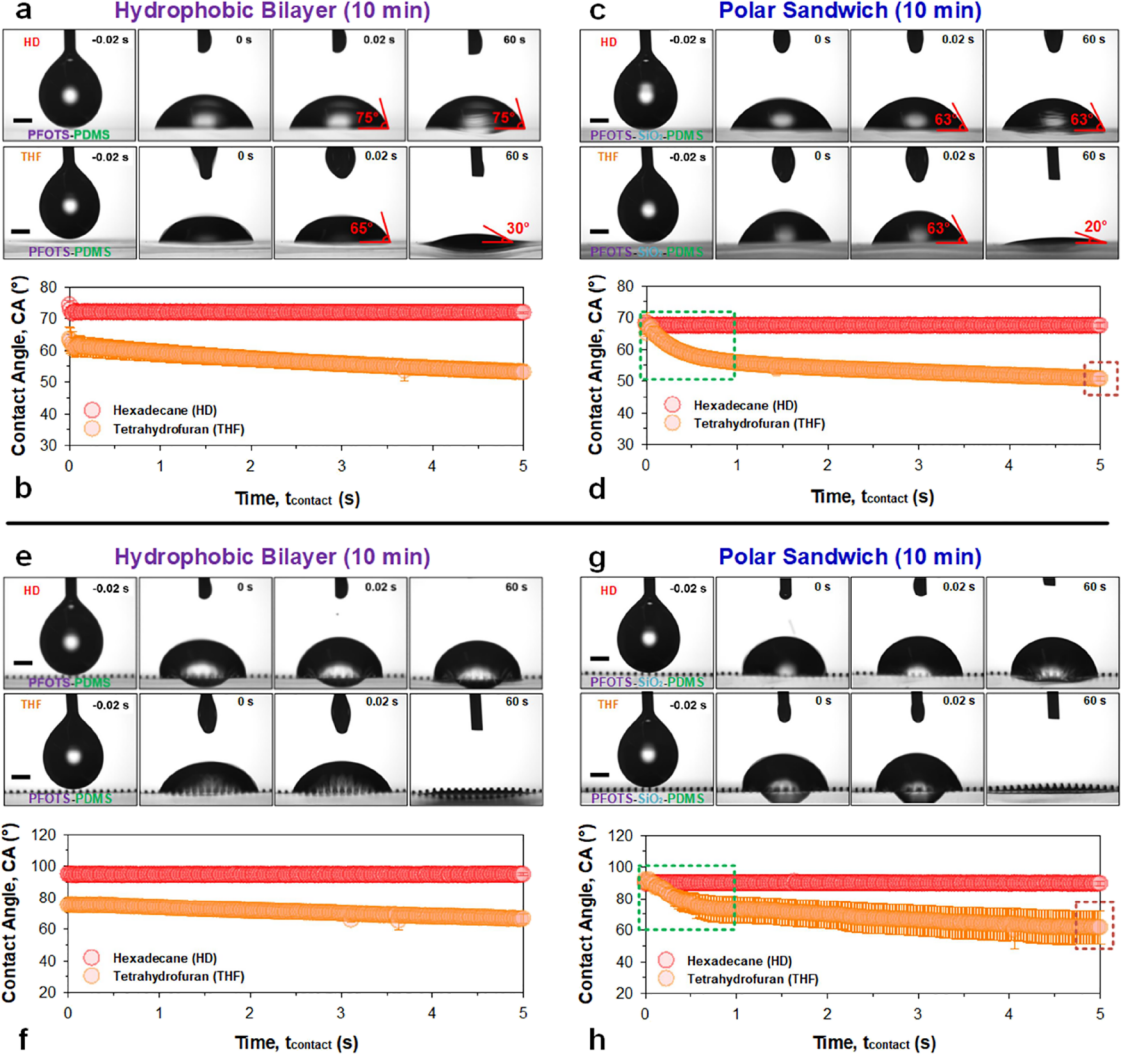
Reactive wetting by polar and nonpolar liquid probes. Wetting on (a–d) macroscopically flat and (e–h) microstructured surfaces with both (a, b, e, and f) nonpolar hydrophobic bilayer (PFOTS–PDMS) and (c, d, g, and h) polar sandwich (PFOTS–SiO_2_–PDMS) configurations. Sessile drop wetting behavior by tetrahydrofuran, THF (orange data, γ_LG_ = 26.4 mN/m), and hexadecane; HD (red data, γ_LG_ = 27.5 mN/m) assesses polarity-induced reactive wetting due to the polar nature of the former. Wetting (*n* = 3, ± SD) was measured over 60 s but plotted for the first (b, d, f, and h) 5 s, where most of the dynamics occurs. Scale bar: 0.5 mm.

On a macroscopically flat nonpolar hydrophobic bilayer (Figure 4a,b) vs the polar sandwich (Figure 4c,d), the nonpolar hexadecane (red data) wets immediately, uniformly, and without initial dynamics. Both establish their final equilibrium contact angles (ca. 71° and ca. 67° respectively) rapidly, without any time-dependent variance (*t*_contact_ = 0.02 to 60 s). The lowered contact angle of nonpolar hexadecane on the polar sandwich is attributed to the increased nonpolar to nonpolar interactions (the sandwich is at least partially dispersive^41,42^). Wetting behaviors lack any observable dynamics, as compared to polar liquids such as water (discussed above) or tetrahydrofuran, as we will see in the following section.

##### Polar Liquid (Flat Surfaces)

With tetrahydrofuran (orange data), wetting occurs almost identically at the beginning for both, albeit at lower contact angles at, ca. 62° for the nonpolar hydrophobic bilayer (Figure 4a,b) and ca. 68° for the polar sandwich (Figure 4c,d). However, the exponential decrease in the contact angle is present only for the polar sandwich. For instance, by *t*_contact_ = 1 s, the nonpolar hydrophobic bilayer was at 59° (Δ = −3°), while the polar sandwich was already at 56° (Δ = −12°). These dynamics are reminiscent of those we have previously observed with water as the wetting probe (Figure 3). This trend continues, while recognizing the volatile nature of tetrahydrofuran, which has a linear contribution beyond *t*_contact_ = 1 s.

##### Nonpolar and Polar Liquids (Microstructured Surfaces)

With the use of model microstructured surfaces, the above trends continue to hold, albeit with higher contact angles and more complex wetting behaviors due to pinning induced by the features. Hexadecane contact angles (red data) on the nonpolar hydrophobic bilayer (Figure 4e,f, top panels) are slightly higher (ca. 95° vs ca. 90°) than those on the polar sandwich (Figure 4g,h, top panels) and remains invariant for both. Tetrahydrofuran contact angles (orange data) start lower for the nonpolar hydrophobic bilayer (ca. 75°, Figure 4e,f, bottom panels) compared to the instantaneously higher values for the polar sandwich (ca. 90°, Figure 4g,h, bottom panels). As observed on flat surfaces, the rate of decrease in the contact angle with tetrahydrofuran on the polar sandwich is much higher. By *t*_contact_ = 1 s, the contact angle reaches ca. 73° (Δ = −17°) for the polar sandwich vs ca. 73° (Δ = −2°) on the nonpolar hydrophobic bilayer.

#### Mechanism: Initial Wetting Dynamics of Polarity-Induced Reactive Wetting

When considering results from all three wetting liquids, (1) water, (2) hexadecane, and (3) tetrahydrofuran, an intriguing wetting trend reveals itself. Notably, whenever both the surface and the liquid are polar in nature, wetting interactions are more dynamic in the first second after liquid contact (Figures 3 and 4, green dotted boxes). The combinations include (1) water and (2) tetrahydrofuran on the polar sandwich surface. A real-time exponential decrease in the contact angle (Figure 3c, blue data and Figure 4d, orange data) occurs despite an initial match in contact angles, even after accounting for linear contributions from evaporation (i.e., for tetrahydrofuran). We do not see such dynamics once either the surface or the liquid is nonpolar (i.e., hydrophobic bilayer with hexadecane or water/tetrahydrofuran). When one partner (surface or liquid) does not possess the ability to achieve polar-to-polar pair interactions, the initial dynamics of polarity-induced reactive wetting disappears. Therefore, wetting-spreading behaviors are strongest on interaction combinations that are both polar (i.e., polar liquid and polar sandwich). The weakest interactions occur when combinations are both nonpolar (i.e., nonpolar liquid with nonpolar surface). Notably, while the legacy wetting models (Zisman,^13^ Owens–Wendt–Rabel–Kaelble (OWRK),^43−45^ and Girifalco-Good^46−48^) do qualitatively support how polar interactions enhances wetting, they are fundamentally equilibrium-state models. Implementation of the OWRK model (see Supporting Information, Table S1) provide close estimates of initial (110° with perfluoroalkyl^49,50^-water) and final wetting (61° with silica^51^-water) states. However, they cannot adequately illustrate these dynamic wetting variations, which we attempt to address in the final section.

#### Complex Reactive Wetting: Drop Spreading-Retracting by Temporal- and Spatial-Competition

**Figure 5:**
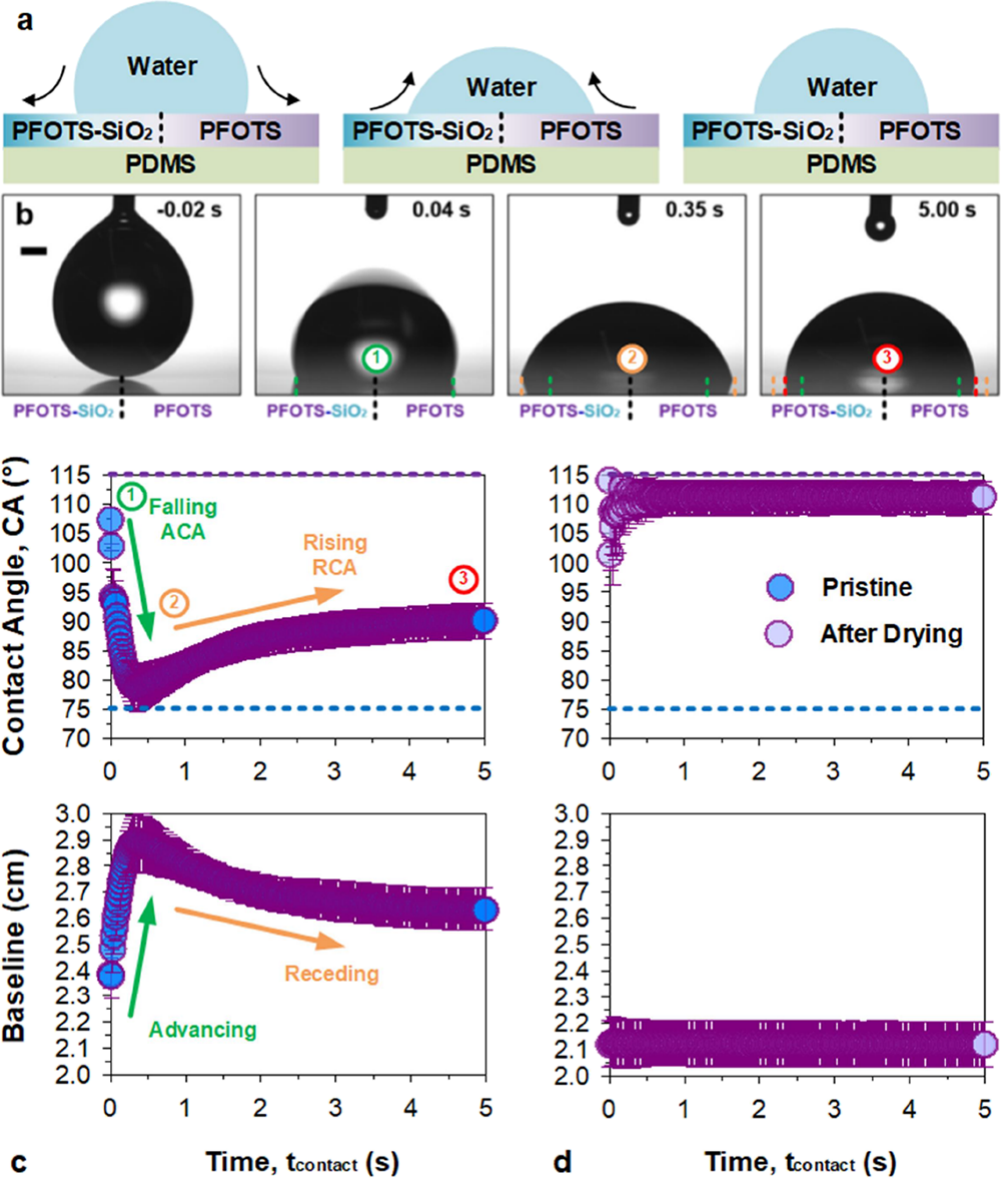
Drop spring phenomenon: sessile drop spreading and retracting. Wetting on an abrupt wetting transition (split-domains), (a) left: wetting-reactive polar sandwich and right: wetting-inert nonpolar hydrophobic bilayer. Sessile drop wetting behavior (over 5 s) by water at the boundary demonstrates a (b and c) dynamic behavior due to the temporally and spatially dynamic wettability. (d) Invariant wettability postwetting confirms the “reactive” to “reacted” nature of the surface. *n* = 5, ± SD (bars). Scale bar: 0.5 mm.

Using our new understanding of polarity-induced reactive wetting, we create a surface with an abrupt wetting transition. This is designed using split-domains with different surfaces (on PDMS). The left half is coated with PFOTS-SiO_2_, forming the polar sandwich. The right half is coated only with PFOTS, forming the hydrophobic bilayer (Figures 5a and S8). The surface was fabricated using PDMS strips as masks. A PFOTS reaction time of *t*_res_ = 10 min was used to confer reactive wetting. XPS analysis was used to confirm that the left and right halves are chemically different (hydrophobic bilayer or polar sandwich). A diffused boundary still likely exists^52^ because of how molecular grafting occurs. By creating split-domains, we introduce wetting–dewetting competition between the two halves, while also likely reducing effective surface pinning by the polar sandwich’s half.

To trigger the wetting dynamics, a drop of water (5 μL) was deposited at the transition. The initial contact angle is high (Figure 5b,c, left panel) but lower than a pure hydrophobic bilayer at ca. 107 ± 2°. Thereafter, it sinks to a minimum of ca. 78 ± 4° (Δ = −28°) at a *t*_contact_ = 0.3 to 0.4 s, which is higher than a polar sandwich. Following which, the contact angle rises to ca. 90 ± 3° (Δ = +12°) in ca. 4.5 s (largest rise within 1.5 s). Visually, the drop sinks (<0.5 s) before springing (1–2 s) upwards, resembling a spring-like behavior. This intriguing wetting–dewetting behavior is achieved without an external energy input, such as magnetism,^17^ thermal,^11,18^ or potential difference (i.e., electrowetting^19^). This is also achieved without the use of autophobic liquids^13−16^ (liquids with surfactants). Lastly, it differs from adaptive wetting^2,3^ or conventional reactive wetting^53,54^ which is driven unidirectionally without reversal.

The drop spring exploits temporal and spatial differences in surface energy to trigger an asymmetric competition between wetting-spreading and dewetting-retracting. The polar sandwich has a lower contact angle limit of ca. 75° (Figure 5c, top panel, blue dashed line), while the hydrophobic bilayer has a higher contact angle limit of ca. 115° (Figure 5c, top panel, purple dashed line). As the drop contacts the boundary, polar interactions from the polar sandwich dominate, initiating drop spreading. The drop shape begins to approach the energetic minima of the solid–liquid–gas interfacial energies for a polar sandwich. As the drop spreads, a significant part of the drop (i.e., the half with the nonpolar, smooth, and nonpinning hydrophobic bilayer) is now in an unoptimal state.

Instead of forming an asymmetrically distorted drop that is energetically unstable, the overall drop symmetry is preserved, and this forces the contact line to retract (Figure 5c, bottom panel). The contact angle arrives at a composited wetting state of ca. 90°. The drop remains axially symmetric throughout the process, with both the left and right contact lines advancing (Figure 5b, green to orange) and receding (Figure 5b, orange to red) simultaneously. Wetting dynamics indicate that contact angles decrease while advancing and increases while receding, opposite to what is conventionally^55^ expected. Here, both spatially- and temporally dependent wetting is important, as a pure spatial gradient would induce asymmetric drop motion^56^ instead of the spring-like behavior. On rare occasions, the surface has been observed to induce limited drop motion at the expense of the equilibrium receding contact angle (see Video M3). This is likely driven by defect-induced asymmetric pinning that disrupts the spatial dependency. An asymmetric drop will experience lateral driving forces.^57^ After drying, wetted locations recover a high contact angle at ca. 111 ± 2° (Figure 5d: top panel). Any nonwetted locations are still able to induce the drop spring effect. In the final section, we explore the temporal dependence of reactive wetting and how it enables such complex wetting-retracting dynamics.

#### Time-Dependent Spreading (τ_s_) and Retracting (τ_r_) Reactive Wetting Modeling

Current models on reactive wetting and spreading kinetics typically illustrate the process in a unidirectional manner (i.e., contact angle decreases to a plateau but does not recover).^58−61^ Recovery after spreading is almost never investigated, and only briefly discussed.^2^ In line with prior formalisms,^2−4^ we attempt now to model both unidirectional and bidirectional reactive wetting (see Supporting Information, MATLAB scripts S1–3).

**Figure 6:**
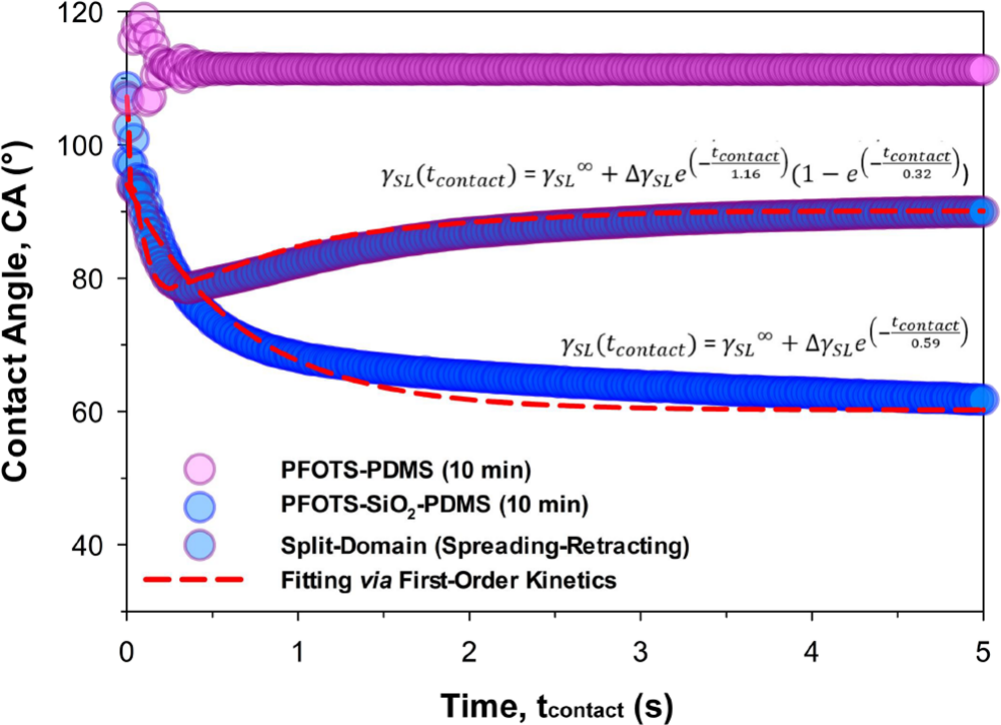
Kinetics of sessile drop spreading and retracting. The upper and lower limits are defined by the surface chemistry (and hence interfacial energy) belonging to each half of the split-domains. The upper limit is defined by the wetting-inert hydrophobic bilayer (PFOTS–PDMS, purple). The lower limit is defined by the wetting-reactive polar sandwich (PFOTS–SiO_2_–PDMS, 10 min). Initial changes in contact angles are attributed to vibrations during drop contact-and-detachment. The spring-like drop and its spreading-retracting behavior can be modeled by first order kinetics of reacting and recovering solid–liquid interfacial energy (Δγ_SL_).

For simplicity, we assume that γ_LG_ and γ_SG_ are both invariant. First, we implement this on the unidirectional reactive wetting observed with water (Figure 1 and 3). We define γ_SL_(*t*_contact_) via the apparent contact angle (θ_app_) alongside the interfacial energies, γ_SG_ = 0.019 J/m^62^ and γ_LG_ = 0.072 N/m. The former is an upper estimate for inert perfluoroalkylated surfaces (Figure 6, purple data).^62^ In combination with Young’s equation, , we define first order spreading kinetics for unidirectional reactive wetting based on time-dependent changes in solid–liquid interfacial energy,
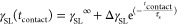
1where  is the final (post reactive wetting) solid–liquid interfacial energy, Δγ_SL_ represents the range of change in γ_SL_ during reactive wetting, and τ_s_ is the characteristic time scale which defines the spreading behavior. τ_s_ may also be interpreted as τ_SL(reactive)_ (reactive adaptation^2^ time scale of γ_SL_).

In this work, we avoid overinterpretation of surfaces fabricated at high *t*_res_ due to significantly increased wicking-spreading behaviors at *t*_res_ > 15 min. This is likely induced by geometrical variations, with contact angles going down to 0° at *t*_res_ = 60 min. As a result, they cannot be appropriately assessed using contact angle measurements, as the computed interfacial energy variation (Δγ_SL_) becomes significantly influenced by geometry. Therefore, we only describe the series via the time scale τ_s_. We ignore the analysis of Δγ_SL_ due to inaccuracies at *t*_res_ > 15 min. Moreover, eq 1 is also valid only until the point of maximum spreading. For the polar sandwich (PFOTS–SiO_2_–PDMS, 10 min), the drops’ contact lines (Figure 6, blue data) do not retract due to surface pinning. However, surface energies postwetting would have also recovered.

Equation 1 is then modified to include time-dependent retraction dynamics. Here, we reintroduce first order kinetics for the recovery of solid–liquid interfacial energy after spreading,
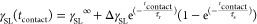
2where τ_r_ is the characteristic time scale, which defines the retraction behavior. τ_r_ may also be interpreted as τ_SL(recovery)_ (recovery adaptation time scale^2^ of γ_SL_). Here,  refers to the proportion of the wetting-reactive surface under the drop that has undergone reactive wetting, as defined by τ_s_. Only the reacted domain experiences the recovery of interfacial energies. In theory, at *t*_contact_ = 0 or ∞, γ_SL_(*t*_contact_) = . Note here that Δγ_SL_ still represents the range of change in γ_SL_ (both increase or decrease). In contrast to eq 1, eq 2 incorporates the possibility of a retracting contact line, representing visual interfacial energy recovery, as we have spatially designed the split-domains to achieve (see the above section).

With Young’s equation, contact angle data (Figure 6, blue data) is fitted to eq 1 (spreading only) or eq 2 (spreading-retracting) to extract key characteristic parameters. First, the reactivity of the polar sandwich, PFOTS–SiO_2_–PDMS, is assessed for its spreading time scale (τ_s_). With a longer PFOTS reaction residence time, *t*_res_, spreading is evidently hastened by wicking. τ_s_ was fitted at an average of 0.59 ± 0.23 and 0.28 ± 0.04 s at *t*_res_ = 10 and 60 min, respectively (*n* = 4, ±SE). Due to the unknown influence by geometrical variations, we exercise prudence when presenting surfaces fabricated under high *t*_res_ (>15 min). We only describe the series via the time scale τ_s_, while acknowledging general inaccuracies in the computed Δγ_SL_. In this work, our analysis focuses on the former (*t*_res_ = 10 min). First, the comparatively smoother variant helps to reduce the influence of geometrical contributions, as evidenced by its hydrophilic but nonwicking nature. Second, the spreading time scale in this variant is also partially entrenched within the retracting dynamics of the spring-like drop (eq 2). Despite efforts to isolate geometrical contributions from interfacial energy variations, readers should be aware that fitting parameters (Δγ_SL_ and τ_s/r_) may still be inadvertently influenced. We present the modeled contact angles (θ, *n* = 4, Figure 6, red dashed line) from the reactive wetting of PFOTS–SiO_2_–PDMS (10 min), alongside experimental data (Figure 6, blue data).

Second, the spreading-retracting behavior of the split-domain is assessed. In this case, we can (optionally) choose to define τ_s_ = 0.59 s, but eq 2 may also be fitted directly (i.e., τ_s_ = 0.32 s). The smaller τ_s_ in the latter is attributed to how spreading was prematurely arrested by recovery-induced retraction. Due to the time-dependent recovery of the solid–liquid interfacial energy (Δγ_SL_), receding of the contact line begins before spreading is completed. Using Young’s equation, contact angle data is fitted to eq 2, giving τ_r_ at ca. 1.16 s (identical regardless of predefining or fitting τ_s_). These τ_s_ and τ_r_ time scales (Figure 6) corroborate prior experimental observations (Figure 5), indicating usefulness of such models. Notably, Δγ_SL_ is negative (spreading-retracting sessile drops) at ca. −0.026 J/m^2^. The negative Δγ_SL_ is iconic of autophobic dewetting, which is typically observed with a rising contact angle upon wetting.^2^ Using Young’s equation, modeled contact angles (θ, *n* = 4, Figure 6, red dashed line) are presented alongside experimental data (Figure 6, blue-purple outlined data).

Future exploration of these basic models should entail complete decoupling of surface energy and geometry^63^ contributions (e.g., incorporating Lucas–Washburn capillary imbibition, etc.). It may also be of interest to investigate reactive wetting in the context of liquid immersion systems,^64,65^ where reactive surfaces under inert liquid mediums are triggered by an immiscible reactive liquid drop. In a liquid–liquid configuration, the smaller viscosity ratios could slow down wetting dynamics, potentially unveiling even more intriguing phenomena.

## Conclusions

Real-world liquids (and surfaces) are often part polar and part nonpolar (dispersive). Therefore, they may experience unique interactions depending on the liquid-surface pair. We show here the design of surface-directed reactive wetting. This was achieved by creating hydrophobic perfluoroalkylated surfaces with embedded polar moieties. We show that the strongest wetting interactions exist for a polar-to-polar liquid to surface configuration. An iconic time-dependent exponential decay in the wetting contact angles is characteristic of the contact-to-spread dynamics. By creating abrupt wetting transitions with split-domains (reactive vs inert), we intertwine time- and spatial- dependent wetting behaviors. Sessile drops contacting these wetting transitions experience highly unique time-dependent spreading and retracting dynamics, which we model using first order kinetics. To this end, we define the spreading (τ_s_) and retracting (τ_r_) time scales that govern the process. The spreading-retracting behavior is attributed to time-dependent relaxations in the interfacial energy (Δγ_SL_), which first decreases at the reactive solid–liquid interface while subsequently recovering. We highlight here the basic design principles of achieving phenomenologically unique surface-directed reactive wetting.

## Supporting Information Available

S1: Scanning electron micrograph of surfaces; S2: Schematized vapor functionalization of perfluoroalkyls; S3: Inert wetting of PFOTS–PDMS vs reactive wetting of PFOTS–SiO_2_–PDMS; S4: X-ray photoelectron spectroscopy on the polar sandwich (PFOTS–SiO_2_–PDMS); S5: EDX analysis of wetted and unwetted domains of a polar sandwich surface; S6: Superspreading of water on PFOTS-SiO_2_ for glass vs PDMS substrates; S7: Ellipsometric analysis of plain PDMS, PFOTS–PDMS, and PFOTS–SiO_2_–PDMS; S8: Visual comparison between plain PDMS, PFOTS–PDMS, and PFOTS–SiO_2_–PDMS; S9: CAH of a control silicon wafer functionalized with PFOTS; Table S1: OWRK Prediction of Water CA; Table S2: Hansen solubility parameters of common probe liquids. (PDF)
Flat surfaces: hydrophobic bilayer vs polar sandwich (MP4)
Structured surfaces: hydrophobic bilayer vs polar sandwich (MP4)
Temporally and spatially contrasted wetting–drop spring effect (MP4)
Unidirectional spreading_wetting fitting script and bidirectional spreading_wetting fitting script with predefined τ_s_ (ZIP)
