# Supplementary material for: Polarity-Induced Reactive Wetting: Spreading and Retracting Sessile Water Drops
Source: Langmuir. 2024 Jun 14;40(26):13562–72. doi: 10.1021/acs.langmuir.4c01085 (PMC11223483; doi:10.1021/acs.langmuir.4c01085)
Supplement: Supplementary file 1 — la4c01085_si_001.pdf [file la4c01085_si_001.pdf]

Supporting Information for

Polarity-Induced Reactive Wetting: Spreading and Retracting Sessile Water Drops

William S. Y. Wong[1], Mariia S. Kiseleva [1], and Abhinav Naga[2]*

Department of Applied Physics, School of Science,
Aalto University, FI-02150 Espoo, Finland[1]

Department of Physics,
Durham University, DH1 3LE Durham, United Kingdom[2]

Keywords: Adaptive and Reactive Wetting, Polar Interactions, Autophobic Dewetting, Surface Controlled, Multi-layer Surfaces

*corresponding author: william.wong@aalto.fi

This file includes:

Supplementary Discussion

- Ellipsometric Measurements of Layer Thicknesses (10 min PFOTS residence time).
- Owens, Wendt, Rabel and Kaelble (OWRK) Models at Static Equilibrium

Supporting Figures S1-9

Table S1

- Hansen Solubility Parameters of Common Probe Liquids.

Supporting Videos M1-3

- 1) Flat Surfaces: Hydrophobic Bilayer *vs.* Polar Sandwich (Water).
- 2) Structured Surfaces: Hydrophobic Bilayer *vs.* Polar Sandwich (Water).
- 3) Temporally and Spatially Contrasted Wetting – Drop Spring Effect.

Supporting MATLAB Scripts S1-3

- 1) Unidirectional Spreading_Wetting Fitting Script.
- 2) Bidirectional Spreading_Wetting Fitting Script with predefined τ_s.
- 3) Bidirectional Spreading_Wetting Fitting Script with undefined τ_s.

Input data (in .xlsx): Column 1: t (s), Column 2: γ_{SL}.

Supplementary Discussion

Spectroscopic ellipsometry for estimation of Layer Thicknesses (vs. PFOTS residence time)

Spectroscopic ellipsometry (SE) is a well-known method for studying thin films, specifically for the evaluation their optical properties alongside thickness and roughness. SE records Ψ (Psi) and Δ (Delta), depicting changes in polarization of the probing beam interacting with the surface as a function of its wavelength.

Modelling: Spectra of PDMS, SiO_2-PDMS, PFOTS-PDMS, and PFOTS-SiO_2-PDMS (residence time 1, 5, 10, 15, 30, and 60 min) were recorded separately (Figure S7a-i). Modelling of PFOTS-SiO_2-PDMS optical properties was complicated due to the very similar refractive indexes of PDMS and SiO_2, at 1.405[1] *vs.* 1.457[2] respectively. This is also further complicated by PFOTS having 1.5 (value estimated from the modelling of SE measurements of a PFOTS layer on silicon substrate). Therefore, optical modelling of the PFOTS-SiO_2-PDMS polar sandwich was limited to a single layer model composed of a mixture of PFOTS-SiO_2 on PDMS substrate. The refractive index of PDMS substrate (1.397) was determined from spectroscopic measurement of plain PDMS substrate and kept constant during the modelling of PFOTS-SiO_2-PDMS. The refractive index and thickness of PFOTS-SiO_2 mixture layer as well as its roughness were modelled using standard Cauchy dispersion equation.[3] The sole layer of SiO_2 was not modelled as a separate layer as SiO_2 solely deposited on the PDMS substrate does not significantly change its optical properties (Figure S7a,b). According to the SE analysis, the sole SiO_2 layer does not increase the roughness of the PDMS, corroborating current SEM results. In contrast to this, PFOTS grown on SiO_2 appears to actively interact during the growth process, resulting in the formation of significantly thicker films as indicated by the larger variation of Δ (Figure S7d-i). Surface roughness was modelled by mixing the top layer with 50 % voids.

Error Considerations: The absolute thickness values obtained for the mixed layer of PFOTS-SiO_2 may have errors induced from the simplified single layer model. In reality, complicated non-uniform structures may evolve with increasing residence time. In addition, the similar

refractive indexes of each sub-component, PFOTS-SiO_2-PDMS, and the strong correlation between both unknown optical constants and film thickness will further reduce sensitivity of SE method. Nonetheless, distinctive differences in the spectra of PFOTS-SiO_2-PDMS (Figure S7d-i) and PDMS without a PFOTS layer (Figure S7b) does confirm the presence of a growing PFOTS-based layer. Increasing variation of Δ (Figure S7d-i) suggests active PFOTS-SiO_2 film growth during increasing residence time.

Results (thickness and roughness): Assuming model validity, the thickness of PFOTS on PDMS (for PFOTS-PDMS) was evaluated as 4.3 ± 0.1 nm whereas for PFOTS-SiO_2 on PDMS (for PFOTS-SiO_2-PDMS) with residence time 1, 5, 10, 15 min (Figure S7d-g), the PFOTS-SiO_2 layer thickness was 43 ± 6, 70 ± 1, 141 ± 2, 226 ± 1 nm, with roughness 45 ± 6, 16 ± 1, 25 ± 1, 12 ± 1 nm respectively. The larger thickness of PFOTS in PFOTS-SiO_2-PDMS can be tentatively explained by the higher reactivity of SiO_2, which promotes the growth of PFOTS layer much more so than on plain PDMS. The roughness of PFOTS-SiO_2-PDMS with 10 min residence time qualitatively corroborates SEM imaging (Figure S1b). The proposed single layer model was in good agreement with experimental data having MSE values lower than 2 for surfaces at 1, 5, 10 min residence time, and a MSE of 6 for 15 min. Accounting for potential errors in the model, as described above, the estimates of PFOTS-SiO_2 thickness within this range demonstrates a trend of increasing film thickness with residence time.

For PFOTS-SiO_2-PDMS with longer residence time 30, 60 min (Figure S7h-i) the thickness was estimated at *ca.* 240, 780 nm with roughness of *ca.* 120, 135 nm respectively. However, the obtained MSE values were higher than 40, indicating that the single layer model is no longer applicable. This suggests that the obtained thicknesses of PFOTS-SiO_2 within this range can only be considered as rough estimates. Nonetheless, the profiles of model and spectroscopic curves are similar, showing oscillation peaks at the same wavelengths.

Owens, Wendt, Rabel and Kaelble (OWRK) Models at Static Equilibrium

$$\gamma_{LV}(1 + \cos(\theta_{inherent}))) = 2[\sqrt{(\gamma_{SV}^{D}\gamma_{LV}^{D})} + \sqrt{(\gamma_{SV}^{P}\gamma_{LV}^{P})}] \quad (1)$$

Table S1. OWRK Prediction of Water Contact Angle on Surfaces

**Surface Energy** / **Species**	$\boldsymbol{\gamma}$ (mJ/m^2)	$\boldsymbol{\gamma_p}$ (mJ/m^2)	$\boldsymbol{\gamma_d}$ (mJ/m^2)	**Contact Angle (°)**
Water	72.8	51	21.8	N.A.
Perfluoroalkylated[4, 5]	19.96	0.33	19.96	108
Silicone[6]	19.8	2.3	17.5	100
Silica[7]	49.4	13.2	36.2	61

Supporting Figures

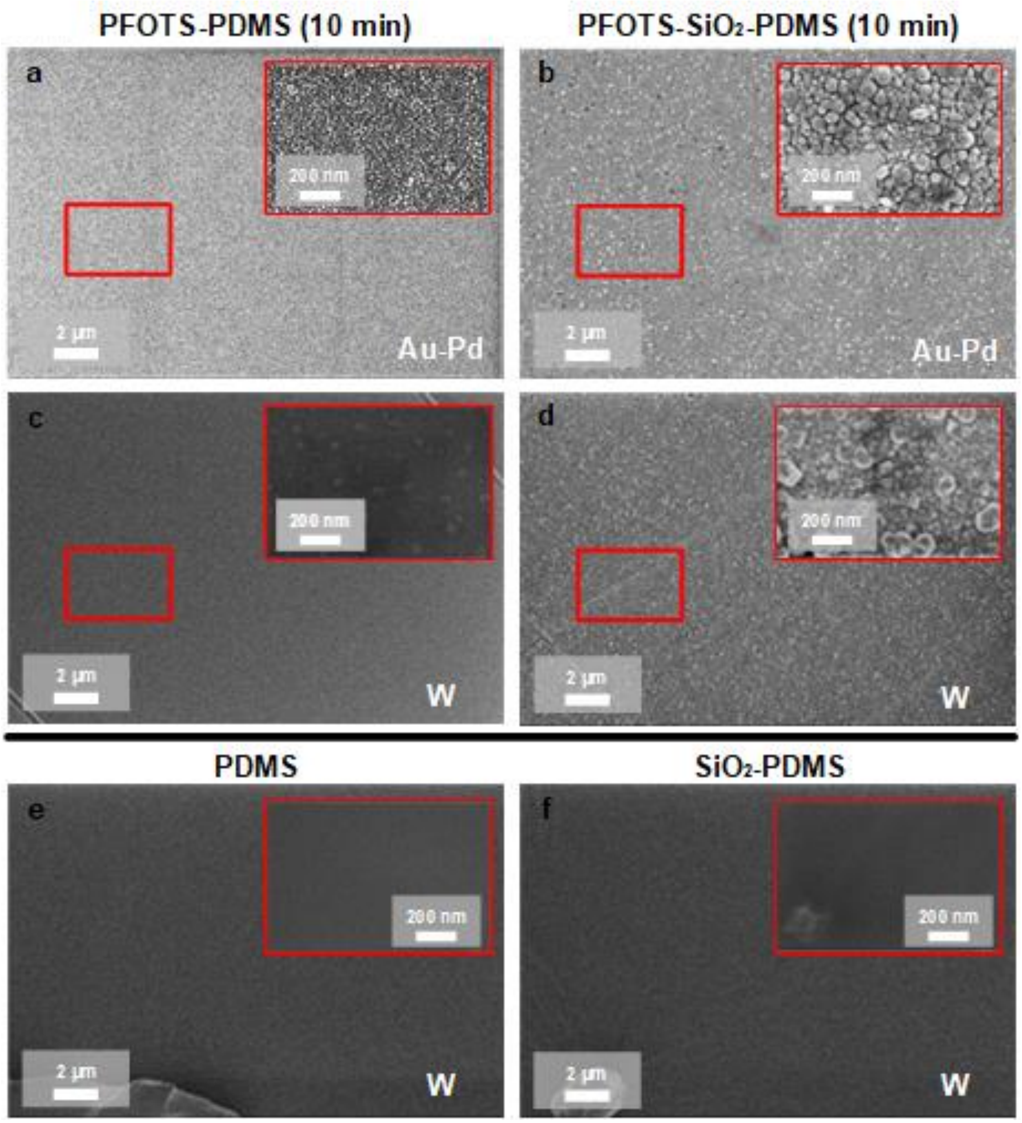

Figure S1. Scanning Electron Micrograph of Quasi-Flat Surfaces with Different Conductive Coatings. a) PFOTS-PDMS and b) PFOTS-SiO_2-PDMS with gold-palladium (AuPd) vs. c) PFOTS-PDMS and d) PFOTS-SiO_2-PDMS with tungsten (W). Evidently, there are a small amount of nanobumps on PFOTS-PDMS, with a diameter of *ca.* 50 nm and below while the nanobumps on PFOTS-SiO_2-PDMS can range up to *ca.* 100 nm. Both e) PDMS and f) SiO_2-PDMS variants are exceedingly smooth, with no surface features except deliberately-introduced point defects that were used in ascertaining surface locations.

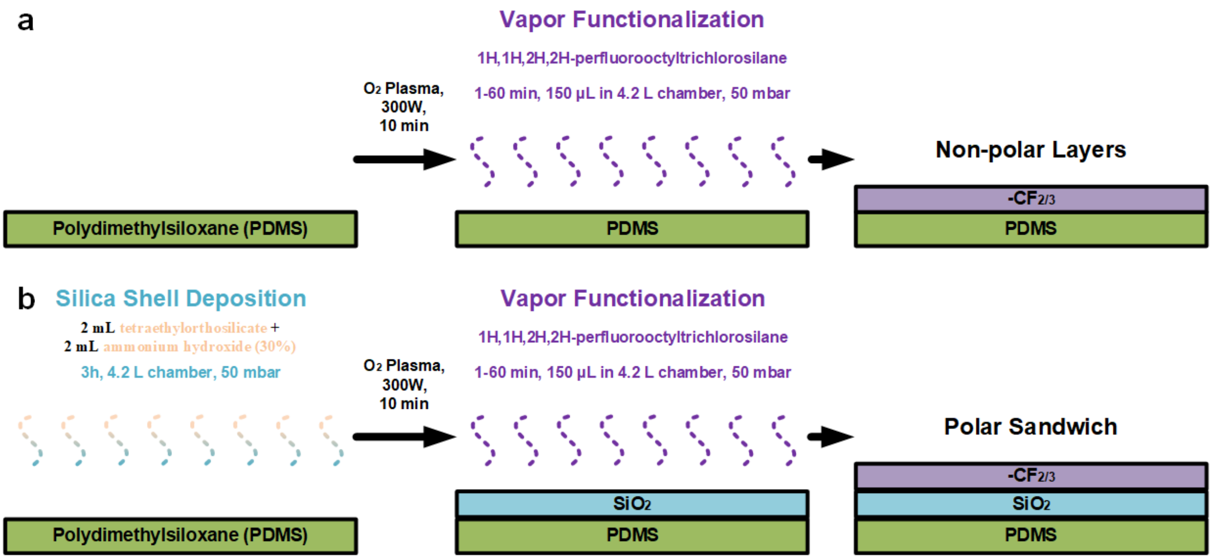

Figure S2. Vapor functionalization of perfluoroalkyls (PFOTS) to create a) pure non-polar or b) polar sandwich surfaces. a) Non-polar: PFOTS on polydimethylsiloxane (PDMS) functionalization creates a surface that is largely non-polar in nature. b) Polar sandwich: PFOTS on silica (TEOS-NH_3) on polydimethylsiloxane (PDMS) is hypothesized to create a polar layer, sandwiched between two non-polar layers. In reality, polar sub-components are found distributed heterogeneously within the perfluoroalkyl layer (See Figure 2, Main Manuscript).

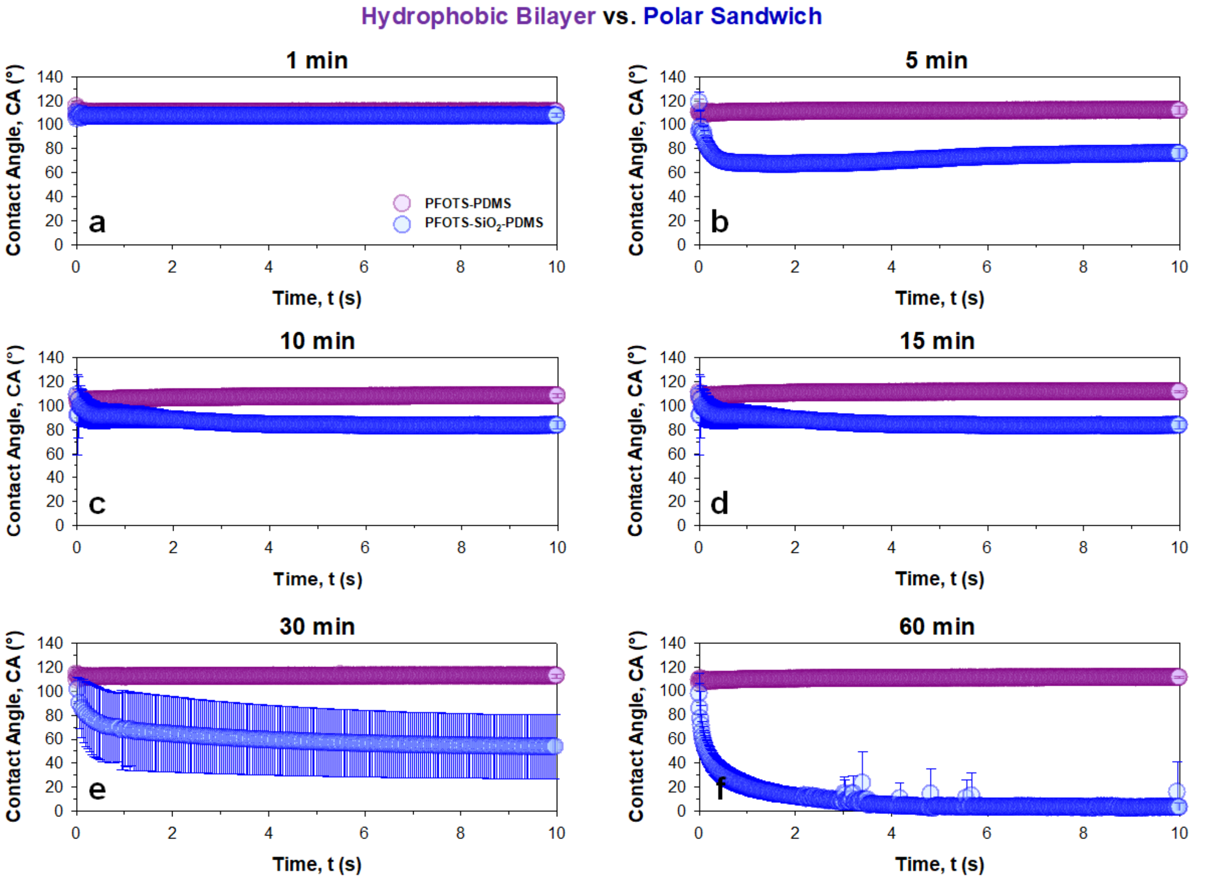

Figure S3. Inert Wetting of PFOTS-PDMS *vs.* Reactive Wetting of PFOTS-SiO$_2$-PDMS. a-f) The sessile drop dynamic contact angle measurements for 10 s after drop contact. The inert hydrophobicity of the bilayer (purple) is stable across all reaction residence time (1 min to 60 min) while the polar sandwich (blue) becomes increasingly wettable. For the latter, the reaction residence time, t_{res}, of b-d) 5 to 15 min shows increased wettability, but a e) strong transition occurs at 30 min, with f) superspreading achieved by 60 min. n = 6, with standard deviations reported.

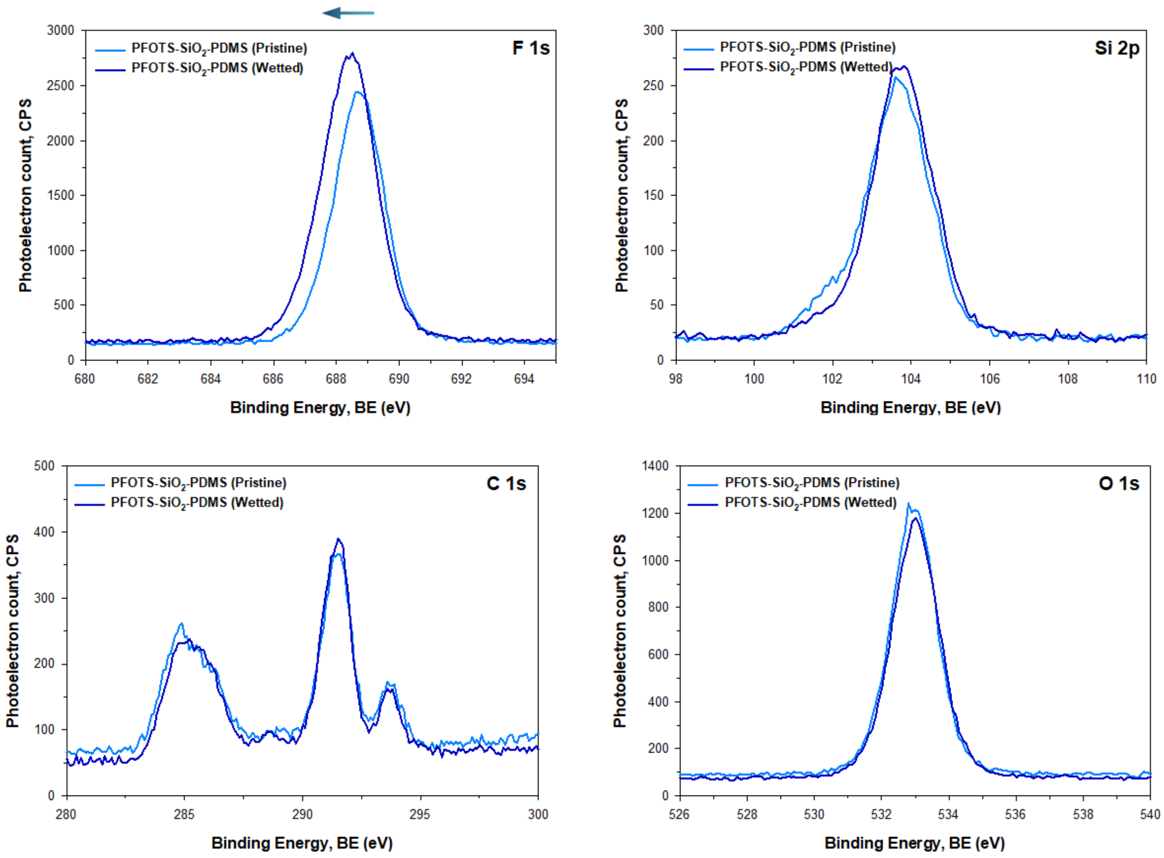

Figure S4. X-ray photoelectron spectroscopy on the polar sandwich (PFOTS-SiO_2-PDMS) at high resolution before (light blue) and after (dark blue) water-wetting, mapping fluorine (F), silicon (Si), carbon (C), and oxygen (O).

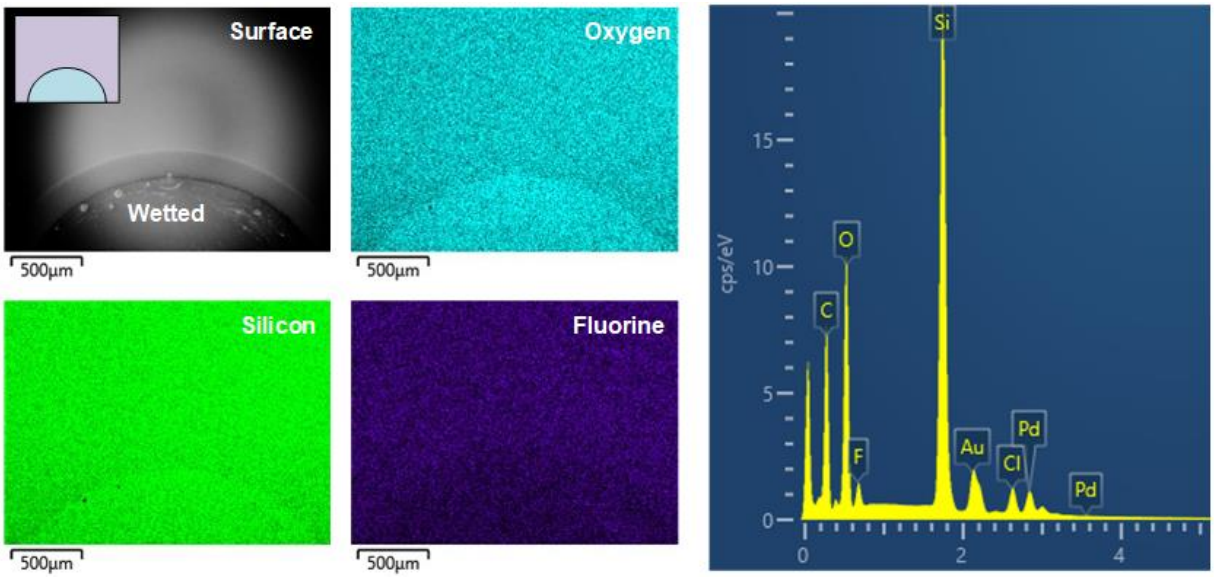

Figure S5. EDX analysis of wetted and unwetted domains of a polar sandwich surface. Key chemical composition comprises of Si, O, and F.

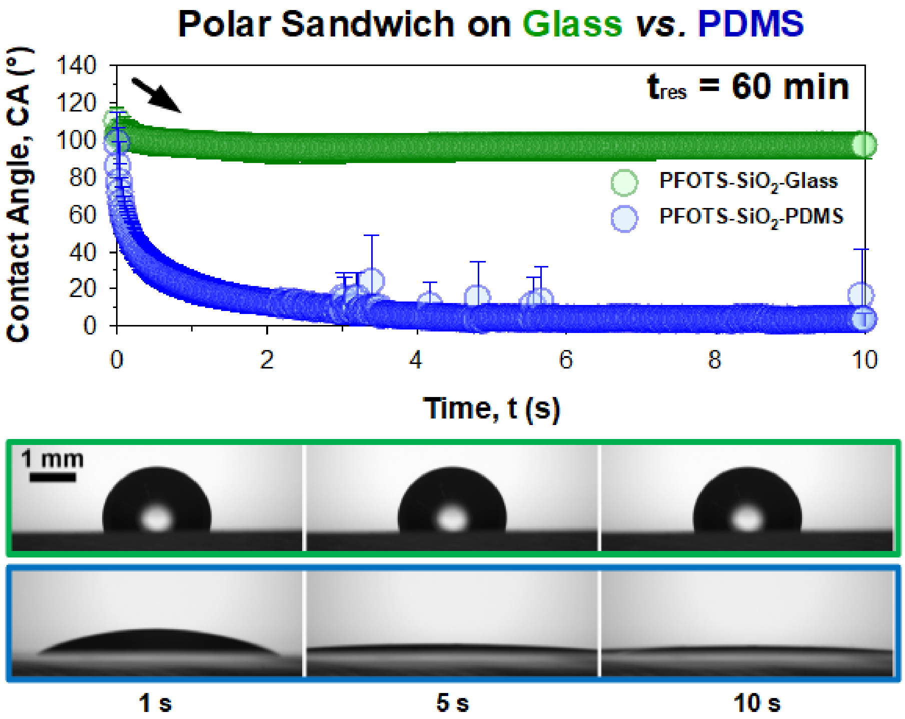

Figure S6. Diminished Superspreading of Water on PFOTS-SiO_2 for substrate glass (green) *vs.* substrate PDMS (blue). The superspreading was suppressed but still evident (115° to 95°).

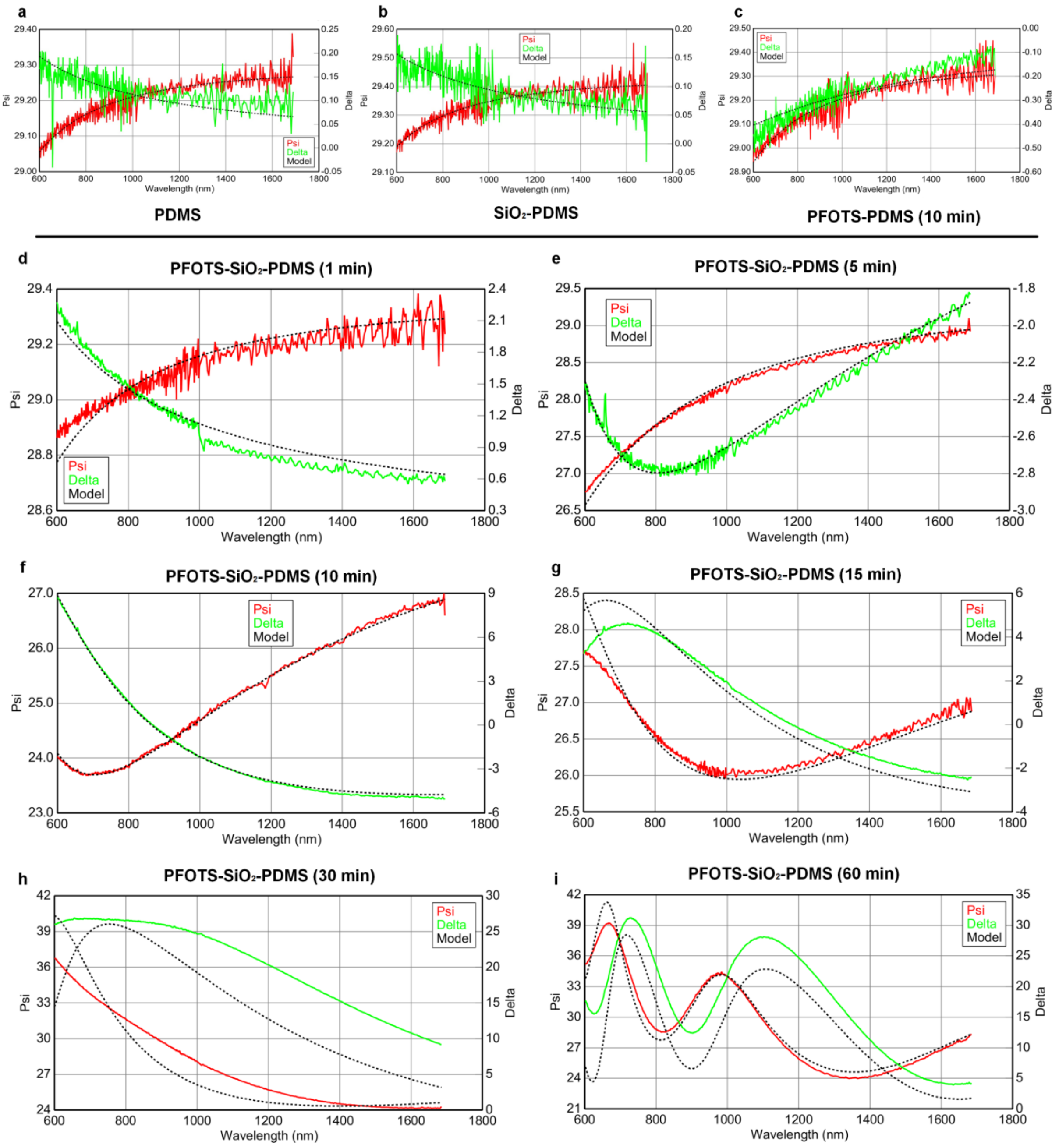

Figure S7. Ellipsometric Analysis of Layers. Spectroscopic ellipsometry data (Psi Ψ and Delta Δ) of a) plain PDMS, b) SiO_2-PDMS, c) PFOTS-PDMS, d-i) PFOTS-SiO_2-PDMS of variable reaction residence time (from 1 min to 60 min). Fitting of 30 min and 60 min variants were not acceptably achieved within reasonable limits. They are therefore classed as “experimentally inaccessible”, as defined in the manuscript, Figure 1d.

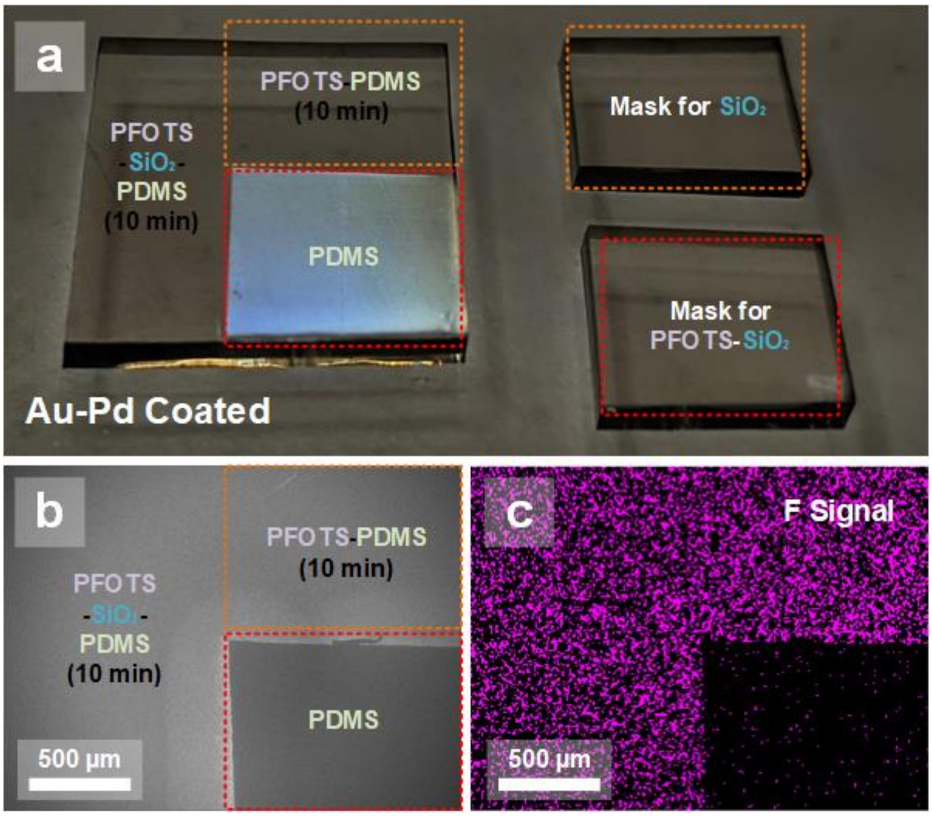

Figure S8. Visual comparison between Plain PDMS, PFOTS-PDMS, and PFOTS-SiO_2-PDMS. a) Grid-coated PDMS surfaces without additional visualization aid. Masks were used to cover domains for SiO_2 and PFOTS-SiO_2 during the multi-step vapor deposition processes. b) Grid-coated PDMS with 10 nm of Au-Pd as visualization aid. Note the shiny surface of plain PDMS (double masked) compared to the undiscernible domains of PFOTS-PDMS (single masked) and PFOTS-SiO_2-PDMS (unmasked). PFOTS likely induced a significant refractive index change, thus allowing optical observation. c-d) SEM-EDX analysis of the grid-coated PDMS, with a focus on the F-signal, showing strong and equal signals on both PFOTS-PDMS (single masked) and PFOTS-SiO_2-PDMS (unmasked) in contrast to plain PDMS (double masked).

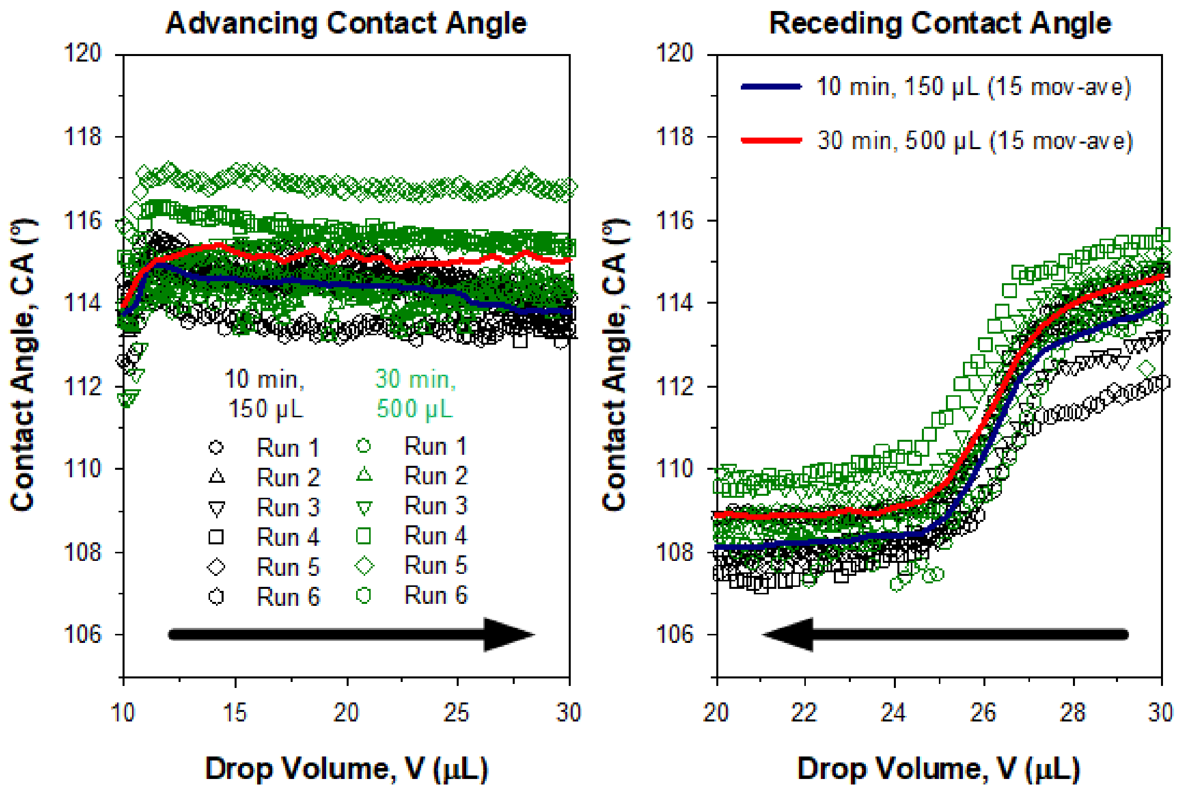

Figure S9. Surface Functionalization of a Control Silicon Wafer with PFOTS. While the use of more PFOTS (500 μL) and longer reaction time (30 min) appears to result in a higher advancing and receding angle (115° and 109°) vs. less PFOTS (150 μL) and shorter reaction time (10 min) with advancing and receding angle (114° and 108°), differences can be considered marginal.

Name	Molecular Formula	Hansen Solubility Parameters		
		δ_d	δ_p	δ_H
N-Methyl-2-pyrrolidine (NMP)	C_5H_9NO	18	12.3	7.2
1,2-Dichloroethane (DCE)	$C_2H_4Cl_2$	19	7.4	4.1
Ethylene glycol (EG)	$C_2H_6O_2$	17	11	26
Hexadecane (HD)	$C_{16}H_{34}$	16.3	0	0
Tetrahydrofuran (THF)	C_4H_8O	16.8	5.7	8
Water	H_2O	15.6	16	42.3

Table S2. The Hansen solubility parameters for literature references[8-11] depicting common instances of polarity-induced wetting and our experimental test liquids. Dispersive ($\boldsymbol{\delta_d}$), polar ($\boldsymbol{\delta_p}$), and hydrogen-bonding ($\boldsymbol{\delta_H}$) parameters are highlighted. Units: MPa$^{1/2}$ (equivalent to joules/cubic centimeter; 2.0455 x (cal/cc)$^{1/2}$) at 25ºC (298.15 K).[12]

References

(1) Graubner, V.-M.; Clemens, D.; Gutberlet, T.; Kötz, R.; Lippert, T.; Nuyken, O.; Schnyder, B.; Wokaun, A. Neutron Reflectometry and Spectroscopic Ellipsometry Studies of Cross-Linked Poly(dimethylsiloxane) after Irradiation at 172 nm. *Langmuir* **2005**, *21* (19), 8940-8946. DOI: 10.1021/la051086i.
(2) Malitson, I. H. Interspecimen Comparison of the Refractive Index of Fused Silica*,†. *J. Opt. Soc. Am.* **1965**, *55* (10), 1205-1209. DOI: 10.1364/JOSA.55.001205.
(3) Tompkins, H. G.; Hilfiker, J. N. *Spectroscopic Ellipsometry: Practical Application to Thin Film Characterization*; Momentum Press, 2015.
(4) Lindner, E.; Arias, E. Surface free energy characteristics of polyfluorinated silane films. *Langmuir* **1992**, *8* (4), 1195-1198. DOI: 10.1021/la00040a029.
(5) Mader-Arndt, K.; Kutelova, Z.; Fuchs, R.; Meyer, J.; Staedler, T.; Hintz, W.; Tomas, J. Single particle contact versus particle packing behavior: model based analysis of chemically modified glass particles. *Granul. Matter* **2014**, *16* (3), 359-375. DOI: 10.1007/s10035-013-0478-9.
(6) Wu, S. Calculation of interfacial tension in polymer systems. *J. Polym. Sci.: Part C: Polym. Symp.* **1971**, *34* (1), 19-30, https://doi.org/10.1002/polc.5070340105. DOI: https://doi.org/10.1002/polc.5070340105 (acccessed 2022/07/25).
(7) Janiszewska, N.; Raczkowska, J.; Budkowski, A.; Gajos, K.; Stetsyshyn, Y.; Michalik, M.; Awsiuk, K. Dewetting of Polymer Films Controlled by Protein Adsorption. *Langmuir* **2020**, *36* (40), 11817-11828. DOI: 10.1021/acs.langmuir.0c01718.
(8) Pan, S.; Guo, R.; Xu, W. Durable superoleophobic fabric surfaces with counterintuitive superwettability for polar solvents. *AIChE J.* **2014**, *60* (8), 2752-2756, https://doi.org/10.1002/aic.14517. DOI: https://doi.org/10.1002/aic.14517 (acccessed 2023/02/21).
(9) Li, B.; Zhang, J.; Gao, Z.; Wei, Q. Semitransparent superoleophobic coatings with low sliding angles for hot liquids based on silica nanotubes. *J. Mater. Chem. A* **2016**, *4* (3), 953-960, 10.1039/C5TA08733C. DOI: 10.1039/C5TA08733C.
(10) Wong, W. S. Y.; Liu, G.; Nasiri, N.; Hao, C.; Wang, Z.; Tricoli, A. Omnidirectional Self-Assembly of Transparent Superoleophobic Nanotextures. *ACS Nano* **2016**, *11* (1), 587–596. DOI: 10.1021/acsnano.6b06715.
(11) Li, W.; Tang, X.; Han, X.; Li, J.; Chu, Y.; Wang, L. Super-alcohol-repellent coatings. *J. Colloid Interface Sci.* **2022**, *613*, 146-154. DOI: https://doi.org/10.1016/j.jcis.2022.01.024.
(12) Hansen, C. M. *Hansen Solubility Parameters: A User's Handbook, Second Edition*; CRC Press, 2007.
